# A Kinetic Response Model for Standardized Regression Analyses of Inflammation-Triggered Hypothermic Body Temperature-Time Courses in Mice

**DOI:** 10.3389/fphys.2021.634510

**Published:** 2021-08-24

**Authors:** Hans H. Diebner, Sören Reinke, Angela Rösen-Wolff, Stefan Winkler

**Affiliations:** ^1^Department of Medical Informatics, Biometry and Epidemiology, Ruhr-Universität Bochum, Bochum, Germany; ^2^Department of Pediatrics, University Hospital Carl Gustav Carus, Technische Universität Dresden, Dresden, Germany; ^3^The Jenner Institute, Nuffield Department of Medicine, University of Oxford, Oxford, United Kingdom

**Keywords:** kinetic response model, mathematical modeling, systems biology, caspase-1 signaling, inflammation, LPS shock, theory-driven nonlinear regression

## Abstract

LPS is frequently used to induce experimental endotoxic shock, representing a standard model of acute inflammation in mice. The resulting inflammatory response leads to hypothermia of the experimental animals, which in turn can be used as surrogate for the severity of systemic inflammation. Although increasingly applied as a humane endpoint in murine studies, differences between obtained temperature-time curves are typically evaluated at a single time point with *t*-tests or ANOVA analyses. We hypothesized that analyses of the entire temperature-time curves using a kinetic response model could fit the data, which show a temperature decrease followed by a tendency to return to normal temperature, and could increase the statistical power. Using temperature-time curves obtained from LPS stimulated mice, we derived a biologically motivated kinetic response model based on a differential equation. The kinetic model includes four parameters: (i) normal body temperature (*T*_*n*_), (ii) a coefficient related to the force of temperature autoregulation (*r*), (iii) damage strength (*p*_0_), and (iv) clearance rate (*k*). Kinetic modeling of temperature-time curves obtained from LPS stimulated mice is feasible and leads to a high goodness-of-fit. Here, modifying key enzymes of inflammatory cascades induced a dominant impact of genotypes on the damage strength and a weak impact on the clearance rate. Using a likelihood-ratio test to compare modeled curves of different experimental groups yields strongly enhanced statistical power compared to pairwise *t*-tests of single temperature time points. Taken together, the kinetic model presented in this study has several advantages compared to simple analysis of individual time points and therefore may be used as a standard method for assessing inflammation-triggered hypothermic response curves in mice.

## 1. Introduction

Inflammation is a complex reaction of the immune system, typically induced by damage- or pathogen associated molecular patterns (DAMPs and PAMPs) and orchestrated by different signaling pathways. Physiologically, inflammation contributes to pathogen clearance and tissue repair. As inflammation is potentially detrimental to the host, pro-inflammatory signaling is controlled by various checkpoints of the respective signaling pathways. Besides its physiological role, dysregulated pro-inflammatory signaling is known to contribute to common diseases like atherosclerosis, rheumatoid arthritis or sepsis, and an increasing number of rare diseases such as periodic fever syndromes (Berghe et al., 2014). In order to unveil new therapeutic targets against inflammation-driven diseases, detailed understanding of inflammatory pathways involved is crucial.

Transgenic mice are a powerful tool to study pro-inflammatory signaling pathways and potential therapeutic interventions in a model organism. The LPS shock model is frequently used in mice to analyze acute inflammation *in vivo* and historically, uses mortality of animals as experimental endpoint. High LPS doses (around 50*mg*/*kg*) typically induce early death of WT animals within 24 h (Kayagaki et al., 2011; Orning et al., 2018), while lower LPS doses may also lead to substantial mortality, but typically at later time points of 24–120 h (Saito et al., 2003; Vandendriessche et al., 2014; Mei et al., 2018). LPS-induced hypothermia is also a well-known surrogate for the severity of systemic inflammation and illness in mice which has been used extensively as endpoint for LPS shock models (Ochalski et al., 1993; Vlach et al., 2000; Saito et al., 2003; Nold et al., 2010; Cauwels et al., 2013; Berghe et al., 2014; Vandendriessche et al., 2014; Mei et al., 2018; Reinke et al., 2020). If the LPS dose is titrated precisely, recovery of the animals from hypothermia—and therefore from systemic inflammation—can be analyzed as well. Additionally, this approach allows the investigation of secondary outcomes like serum cytokines or histological analysis of organs at predefined time points. In order to investigate potential differences of systemic inflammatory responses between experimental groups, most published studies favor single time point comparisons although entire temperature-time series would be available (Vlach et al., 2000; Saito et al., 2003; Nold et al., 2010; Cauwels et al., 2013). This approach, however, leaves substantial information aside. If the body temperature is repeatedly measured over time, the resulting temperature-time curve can be analyzed using mathematical modeling.

The caspase-1 pathway plays a pivotal role in pro-inflammatory signaling induced by the innate immune system (Winkler and Rösen-Wolff, 2015). Activation of caspase-1 itself is initiated by a large multi-protein complex called the inflammasome. When activated following LPS stimulation *in vivo*, this pathway—besides others—leads to systemic inflammation and hypothermia of the experimental animals. Enzymatically active caspase-1-induced inflammation is mainly based on the secretion of the pro-inflammatory cytokines IL−1β and IL-18. This pathway is missing in *Casp*1*KO* and enzymatically inactive Casp1 C284A mice. Interestingly, enzymatically inactive Casp1 C284A mutant activates inflammation via a non-canonical, RIP2-dependent and TNFα mediated pathway (Reinke et al., 2020). The genotypes analyzed in this study were selected from a larger set of experiments from our group (Reinke et al., 2020). As these genotypes modify the LPS-dependent inflammatory and hypothermic response we exemplarily used the corresponding datasets to test our novel kinetic response model.

Here, we introduce a novel kinetic response model based on a dynamic differential equation accurately fitting to measured body temperature-time courses resulting from LPS-induced acute inflammation. The kinetic model is composed of biologically interpretable components, leads to a high goodness-of-fit and provides enhanced statistical power compared to single time point comparisons. Therefore, it may be established as a standard kinetic model for assessing inflammation-triggered hypothermic response curves in mice.

## 2. Methods

### 2.1. Experimental Model

#### 2.1.1. Mice

Wild-type *C*57*BL*/6*N* (*Casp*1*WT*) mice were purchased from Charles River and bred in-house. *Casp*1*KO* and *Rip*2*KO* mice were described previously (Ruefli-Brasse et al., 2004; Case et al., 2013). Casp1 C284A mice were generated using conditional gene targeting (see Reinke et al., 2020 for details). Male and female mice 9–14 weeks of age were used for the *in vivo* experiments. The following 6 genotypes were analyzed (*genotype*:*n*): *Casp*1*WT*:56, *Casp*1*WT*/*Rip*2*KO*:17, *Casp*1*KO*:57, *Casp*1*KO*/*Rip*2*KO*:14, *Casp*1*C*284*A*:64, *Casp*1*C*284*A*/*Rip*2*KO*:29. All animal procedures were performed according to institutional guidelines and in accordance with the Landesdirektion Sachsen.

#### 2.1.2. *In vivo* LPS Application

10*mg*/*kg* LPS (E.coli 0111:B4, Invivogen) was injected intraperitoneally. The ambient temperature was actively controlled and set to $T_{a}=23^{∙}C$ for all experiments. Body temperature of mice was measured every 6*h* for 24*h* using a rodent rectal temperature probe (*t*∈{0, 6, 12, 18, 24*h*}). For all *in vivo* studies age and sex matched animals of the designated genotypes were used (see Reinke et al., 2020 for details).

### 2.2. Kinetic Model

To adequately model the observed temperature-time courses *T*(*t*), two mechanisms have to be considered. Firstly, LPS stimulation, approximately in form of a bolus, induces inflammation thereby leading to a perturbation from normal body temperature, *T*_*n*_. Secondly, a repair process regulates the temperature back to *T*_*n*_.

#### 2.2.1. Temperature Autoregulation

Temperature autoregulation asymptotically adjusts temperature to a stable body temperature, *T*_*n*_. Specifically, it is assumed that in absence of LPS treatment a self-regulatory process in form of a logistic dynamics maintains an asymptotically stable temperature:

(1) $$\begin{array}{r} \frac{dT\left(t\right)}{dt}=rT\left(t\right)\left(1-\frac{T\left(t\right)}{T_{n}}\right). \end{array}$$

**Figure 1:**
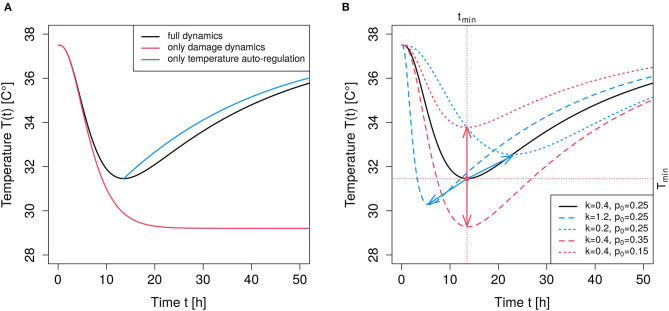
Illustration of parameter impact. **(A)** The black curve shows the time course of the full dynamics given by Equation (5) with *s* = 3, $T_{n}=37.5^{∙}C$, $r=0.04\frac{1}{h}$, $k=0.4\frac{1}{h}$, *p*_0_ = 0.25, *T*(*t* = 0) = *T*_*n*_. The red curve results from the damage part alone, i.e., *r* = 0.0 and all other parameters as for the full dynamics. The blue curve results from setting *p*_0_ = 0 when the point of minimum temperature is reached in the full dynamics model, and shows the effect of temperature regulation alone. **(B)** Black curve as in the left panel (full dynamics). The red curves result upon changing the value of *p*_0_, i.e., a larger *p*_0_ = 0.35 yields the dashed red curve, a smaller *p*_0_ = 0.15 yields the dotted red curve, thus, changing *p*_0_ leads to a vertical shift of the temperature minimum (see red arrows). The blue curves result upon changing the value of *k*, i.e., a larger $k=1.2\frac{1}{h}$ gives the dashed blue curve and a smaller $k=0.2\frac{1}{h}$ leads to the dotted blue curve. Increasing the value of *k* lowers the minimum and shifts the minimum to an earlier time (see blue arrows).

This logistic dynamics is considered as an adequate phenomenological description to an effective process of autoregulation. The two free parameters, *r* and *T*_*n*_ are kinetically interpretable: *T*_*n*_ is the asymptotically stable normal body temperature and *r* quantifies the stabilizing force. This approach is in line with recent findings that the classical way of modeling thermal regulation in form of a linear dynamics appears to be oversimplified (Boldrini et al., 2018). In comparison to the linear dynamics, the logistic dynamics adds an inflection point in the hypothermic case, i.e., for a drop of temperature below *T*_*n*_. Figure 1A shows an exemplary time course (blue curve).

#### 2.2.2. Hypothermic Response Upon LPS Stimulation

In sub-thermoneutral ambient temperatures, mice respond to LPS with hypothermia, which can be used as surrogate for the severity of systemic inflammation (Rudaya et al., 2005; Steiner et al., 2011). Thus, the perturbation process, *p*(*t*), gives rise to a temperature drop −*p*(*t*)*T*(*t*) yielding the combined dynamics

(2) $$\begin{array}{r} \frac{dT\left(t\right)}{dt}=rT\left(t\right)\left(1-\frac{T\left(t\right)}{T_{n}}\right)-p\left(t\right)T\left(t\right). \end{array}$$

In a first approximation, we assume that injection of LPS during a very short time period (bolus) leads to an initial maximum perturbation *p*_0_ and a subsequent linear clearance dynamics with kinetic parameter *k*, thus

(3) $$\begin{array}{r} \frac{dp\left(t\right)}{dt}=-kp\left(t\right)\text{with}p\left(t=0\right)=p_{0}k. \end{array}$$

Using the explicit solution of Equation (3), i.e., an exponential decay function, leads to the model for the hypothermic process given by,

(4) $$\begin{array}{r} \frac{dT\left(t\right)}{dt}=rT\left(t\right)\left(1-\frac{T\left(t\right)}{T_{n}}\right)-p_{0}k\exp\left(-kt\right)T\left(t\right). \end{array}$$

#### 2.2.3. Clearance Dynamics

The clearance dynamics in form of an exponential function *p*_0_*k*exp(−*kt*) can be interpreted as a process with exponentially distributed clearance times with median (half life) given by ln(2)/*k*. Thus, rate constant *k* has dimension $\left[\frac{1}{t}\right]$, whereas parameter *p*_0_ is a dimension-free multiplier which defines the initial strength of perturbation. The spatial extensions of real physiological systems are often modeled in form of *s* serially connected transition compartments, each characterized by an exponential decay, finally leading to gamma-distributed residence times (Sun and Jusko, 1998). Therefore, a Gamma distribution function $f\left(t,s,k\right)=\frac{k^{s}t^{s-1}}{\left(s-1\right)!}\exp\left(-kt\right)$ with shape parameter *s* may be substituted for the exponential distribution, i.e.,

(5) $$\begin{array}{r} \frac{dT\left(t\right)}{dt}=rT\left(t\right)\left(1-\frac{T\left(t\right)}{T_{n}}\right)-p_{0}f\left(t,s,k\right)T\left(t\right). \end{array}$$

The original exponential distribution can then be reproduced by setting *s* = 1. An exemplary time course of the damage dynamics alone (red curve) in comparison to the full dynamical behavior (black curve) is illustrated in Figure 1A. Without the first temperature autoregulation term in Equation (5) the body temperature would asymptotically reach a stable deviation from *T*_*n*_. Such a decoupled dynamics is not observed under physiological conditions and mentioned here merely to clarify the contributions of the two superimposed mechanisms.

#### 2.2.4. Dynamic Features of the Model

After the LPS stimulation, the temperature drops in a sigmoidal manner. This deviation is increasingly balanced by an impact of autoregulation and a simultaneously decaying force of perturbation. We refer to the period from the initiation of inflammation up to the time *t*_*min*_ when temperature is minimum as the damage phase. The period from *t*_*min*_ onwards is referred to as the repair phase.

An illustration of the dynamic behavior is depicted in Figure 1B. By solely changing the value of *p*_0_ in Equation (5) while keeping all other parameters at a constant value, leaves the time point, *t*_*min*_, of maximum deflection, *T*_*min*_, invariant but modulates *T*_*min*_ (illustrated by the red arrows in Figure 1). In contrast, changing the value of *k* only, essentially shifts the time point, *t*_*min*_, of maximum deflection, *T*_*min*_, and, in addition, changes the maximum deflection (illustrated by the blue arrows in Figure 1). Thus, the temperature perturbation related to the magnitude of damage is mainly determined by parameter *p*_0_, whereas clearance parameter *k* crucially determines the duration of the perturbation, which corresponds to an intuitive understanding of “clearance.”

It should be noted that a reliable estimation of all involved parameters requires a time series recorded beyond the minimum point *t*_*min*_. Otherwise, when using records only up to *t*_*min*_, the effective summary process hampers an independent estimation of *r*, *p*_0_ and *k*. The damage phase is most informative for the evaluation of *p*_0_ and *k*, whereas the repair phase is most relevant for the estimation of *r* (and *T*_*n*_, if necessary).

The kinetic parameters, particularly the parameters *p*_0_ and *k*, may actually depend on the LPS dosage. LPS-induced inflammation triggers a cascade of pro-inflammatory and inhibitory signaling via cytokine production, which gives rise to complex physiological regulation processes (Dobreva et al., 2021). Therefore, our model has to be conceived as a phenomenological “summary process” with different kinetic processes aggregated into a manageable number of parameters.

### 2.3. Regression and Model Selection

The kinetic model introduced in the previous section is numerically integrated using the *deSolve*-package (Soetaert et al., 2010) and fitted to the observed temperature-time courses using the package *drc* (Ritz et al., 2015) of the statistical programming language *R* (R Core Team, 2021). This package allows to handle prediction models in a straightforward way. For each of the 4 parameters, *r*, *T*_*n*_, *p*_0_, and *k*, it is possible to independently define whether or not it depends on a predictor such as the genotype or an administered inhibitor (as in Reinke et al., 2020), respectively. The *drc*-package allows for a pairwise comparison of levels of the predictor per parameter in a comfortable way. The routine is based on the least squares method. Since the logarithms of the repeated measurements of the response values (temperature) appear to be approximately normally distributed, we used logarithmized temperature values for the least squares fits. In addition, parameter estimates have been accomplished at the log-level. This is crucial when assessing the reported confidence intervals supplied by the drc-routine for the de-logarithmized estimates. The model selection process is based on likelihood ratio tests. In order to demonstrate the discriminative power and superior goodness-of-fit of the kinetic model-based approach, comparisons with common statistical methods as *t*-tests (applied to temperature measurements at single time-points or to the area under the curve, respectively) and with an analysis of variance (ANOVA) were conducted.

### 2.4. Comparison Across Genotypes

Pairwise comparisons of genotypes are performed as following. Firstly, the likelihood (goodness of fit) from fitting the model to data with full degrees of freedom is calculated. Secondly, after treating a pair of genotypes as if they were one and the same genotype, i.e., after identification of two levels of the predictor, the new likelihood with correspondingly reduced degrees of freedom is calculated. Finally, a likelihood ratio test decides whether to keep the null hypothesis of indistinguishability (with respect to hypothermia) of the two genotypes or to opt for a significant difference (Azzalini, 1996).

## 3. Results

### 3.1. Model Selection

**Figure 2:**
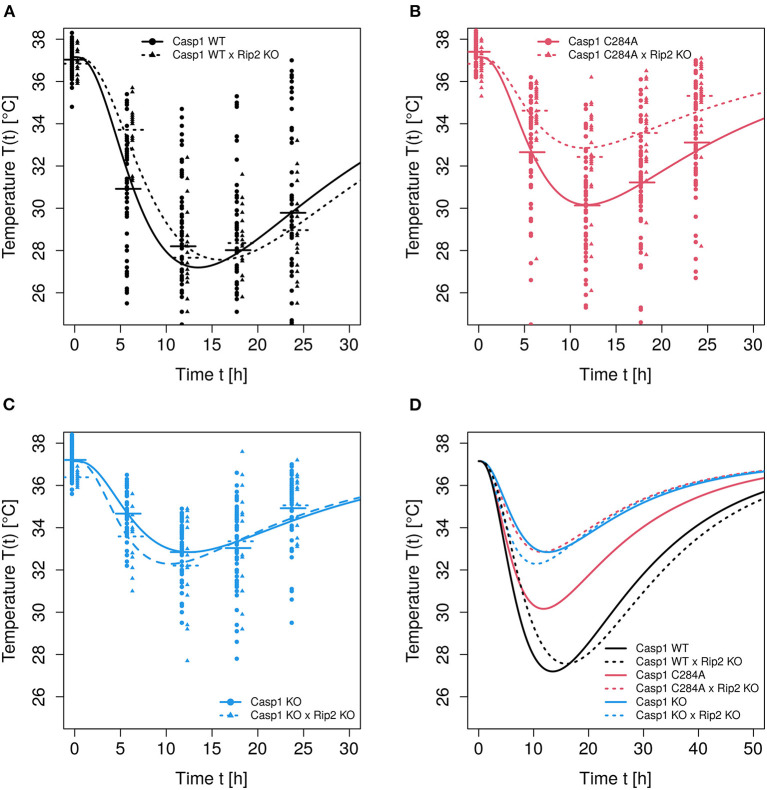
Observed and predicted temperature-time courses. Modeling of temperature-time curves was performed using model Equation (5) with *s* = 3, estimating *k* and *p*_0_ as group specific. **(A–C)** Temperature-time courses (observations plus model predictions) of the respective genotypes *Casp*1*WT*, *Casp*1*C*284*A* and *Casp*1*KO* (each on *Rip*2*WT* or *Rip*2*KO* background). **(D)** All 6 predicted temperature-time courses are shown.

Following LPS stimulation, body temperature-time courses have been measured for six different experimental groups, represented by six levels of the predictor. The model predictions depicted in Figure 2 result from fitting model (Equation 5) to the data, whereby the Gamma distribution shape parameter has been set to *s* = 3. Furthermore, two of the four free parameters, i.e., *k* and *p*_0_, have been stratified for the predictor. Parameters *T*_*n*_ and *r*, to the contrary, have been estimated as averages over the six levels of the predictor. Therefore, 14 kinetic parameters have been estimated. This model is the result of a stepwise model selection procedure:

In a first step, model (Equation 5) with shape parameter *s* = 1 has been fitted to the data with all parameters fully stratified for the predictor, thus, 24 parameters have been estimated. In a common notation the corresponding prediction formula reads *pred*_0_ = (*T*_*n*_~*genotype, r*~*genotype, k*~ *genotype, p*_0_~*genotype*).

In the second step, an average value for *T*_*n*_ was estimated (*pred*_1_ = (*T*_*n*_~1, *r*~*genotype, k*~*genotype, p*_0_~*genotype*)), thereby reducing the degrees of freedom by 5. A likelihood ratio test (*pred*_1_ vs. *pred*_0_) gives *p* = 0.82. As expected, the asymptotically stable body temperature *T*_*n*_ does not depend on the genotype. An additional reduction of 5 degrees of freedom by averaging *r* over the 6 genotypes (*pred*_2_ = (*T*_*n*_~1, *r*~1, *k*~*genotype, p*_0_~*genotype*)) yields *p* = 0.98 (*pred*_2_ against *pred*_0_). Thus, the stabilizing force *r* of temperature autoregulation does not depend on the genotype if *s* = 1.

Proceeding with the reduction of degrees of freedom by application of the above scheme to parameters *k* (*pred*_3_ = (*T*_*n*_~1, *r*~1, *k*~1, *p*_0_~*genotype*) vs. *pred*_0_, *p* = 0.0028) and *p*_0_ (*pred*_4_ = (*T*_*n*_~1, *r*~1, *k*~*genotype, p*_0_~1) vs. *pred*_0_, *p*≃10^−12^), indicated that the genotype-dependence of the temperature-time curves is largely determined by parameter *p*_0_ and only slightly by *k*.

Next, we set shape parameter *s* = 2 and run through the same procedure of parameter reduction as before. This leads to the following series of *p*-values: *pred*_1_ vs. *pred*_0_:*p* = 0.86; *pred*_2_ vs. *pred*_0_:*p* = 0.77; *pred*_3_ vs. *pred*_0_:*p* = 0.0003; and *pred*_4_ vs. *pred*_0_:*p*≃0. Models *pred*_0_ through *pred*_3_ with *s* = 2 fit substantially better compared to the corresponding model using *s* = 1. Specifically, a comparison of models (*pred*_2_, *s* = 1) and (*pred*_2_, *s* = 2) yields deviation 2log(*LR*) = 54.8 which corresponds to *p* = 6.9·10^−14^ of the χ^2^(*DF* = 1)−distribution as an *ad hoc* precision criterion for the shape parameter in favor of *s* = 2. Model *pred*_4_ is neither for *s* = 1 nor for *s* = 2 a relevant option.

A further improvement of the goodness-of-fit can be achieved by setting *s* = 3. The same procedure of parameter reduction as before leads to: *pred*_1_ vs. *pred*_0_:*p* = 0.85; *pred*_2_ vs. *pred*_0_:*p* = 0.031; *pred*_3_ vs. $pred_{0}:p=8\cdot 10^{-6}$; and *pred*_4_ vs. *pred*_0_:*p*≃0. A comparison of models (*pred*_1_, *s* = 3) and (*pred*_1_, *s* = 2) yields deviation 2log(*LR*) = 7.2 corresponding to *ad hoc* *p* = 0.004 in favor of *s* = 3. Parameter *r*, which determines the strength of temperature autoregulation, significantly depended on the genotype. However, due to the correlation of *k* and *r*, this weak significance is kinetically irrelevant. Thus, we kept the model with prediction formula *pred*_2_ and shape parameter *s* = 3 as reference for the following analyses. The predicted temperature-time courses resulting from this reference model are depicted in Figure 2.

Choosing a shape parameter *s* = 4 yields a slightly worse fit in terms of the likelihood, although not significant (*ad hoc* *p* = 0.2), for the full parameter model leaving *s* = 3 as an optimal choice for the shape parameter. Furthermore, the shape parameter *s* = 3 represents the optimal choice for the majority of experimental settings examined in the study of Reinke et al. (2020) (data not shown). Since shape parameter *s* is integer, neither a profile likelihood nor a Hessian (covariance) matrix can be used to estimate confidence intervals. Therefore, the chosen *ad hoc* likelihood ratio test based on one degree of freedom is here conceived as an alternative assessment of precision of the estimated integer value of *s*. It has been found in a different context (see Ganusov and Auerbach, 2014; Ganusov and Tomura, 2018) that fitting gamma distribution functions with shape parameters *s*>1 to transition processes exhibiting delays may indeed slightly improve the goodness of fit, however leading to unreliable estimates of average transit times whenever there is evidence that the distribution of transit times deviates from exponential or gamma. Although this result has to be kept in mind, it is not a strong limitation in the given context for two reasons. Firstly, we observe an overwhelming improvement of goodness-of-fit by switching from exponential to gamma, secondly, for the comparisons of pairs of temperature time courses, the goodness-of-fit has priority over the precision of kinetic parameters. The usage of a Gamma distribution with *s* = 3 leads to a delayed start of the damage phase with a roughly 2 h long “plateau” of the curve at the beginning (see Figure 2) and a narrower subsequent temperature “valley” when compared to the pure exponential clearance function. For a better visual assessment of how observed individual time series relate to the prediction curve, we refer to Supplementary Figure 1.

### 3.2. Parameter Estimations: *p*_0_ and *k* Are Genotype-Dependent

**Figure 3:**
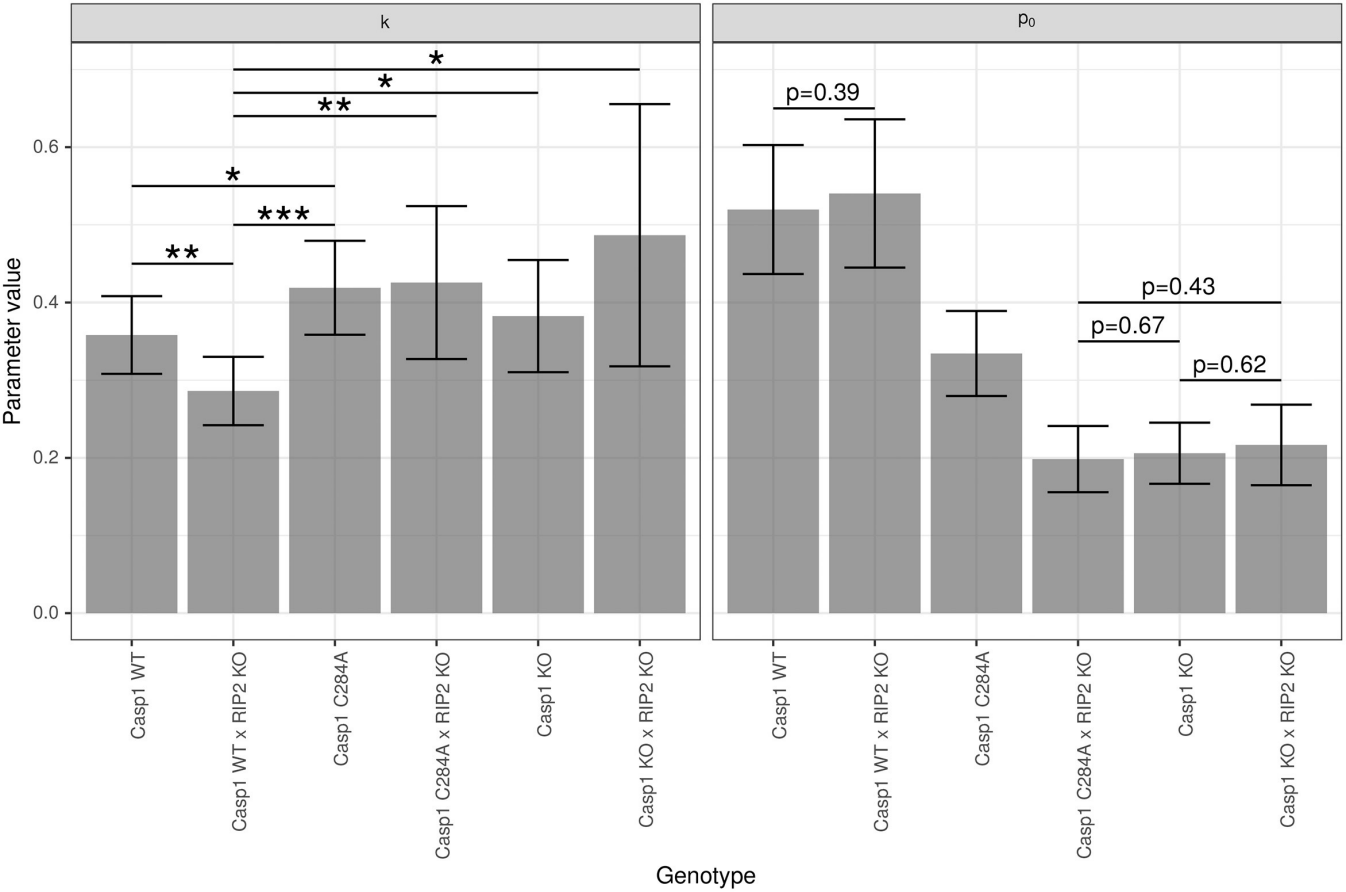
Group-specific estimates of parameters *k* and *p*_0_. Estimation of the parameters $k\;\left[\frac{1}{h}\right]$ and *p*_0_ (dimension-less number) was performed using model (Equation 5) with *s* = 3. Data are presented as mean (±95% confidence intervals). Statistical analysis was performed using two-sided, pairwise *t*-tests. All *p*-values are adjusted for multiple testing using the Benjamini-Hochberg algorithm. Only the 6 significant differences for *k* (0 < *** ≤ 0.001 ≤ ** ≤ 0.01 ≤ * ≤ 0.05) and the 4 non-significant differences for *p*_0_ are marked.

For the given experimental setting, the differences of the temperature-time courses are mainly determined by the genotype-dependent damage strength *p*_0_, although the impact of genotype-dependent clearance rate *k* cannot be neglected completely. The significances resulting from pairwise comparisons of genotype-dependent values of *p*_0_ (see Figure 3) confirm the assessment from a visual inspection of the predicted time courses shown in Figure 2. Experimental settings investigated in the study of Reinke and colleagues (Reinke et al., 2020) lead to clearance rates which are even less dependent on the genotype (data not shown). In these cases the overall distinction of temperature-time courses solely depends on the differences in genotype-dependent damage strength *p*_0_. However, for the data analyzed in this study a moderate impact of the genotype on the clearance parameter *k* is detectable (see Figure 3). Although there is no difference in the parameter *p*_0_ (which affects the maximum temperature drop) between *Casp*1*WT* and *Casp*1*WT*/*Rip*2*KO* animals, differences in parameter *k* (related to LPS clearance) lead to a shift of the time at minimum temperature (see Figure 2), thereby contributing with *p* = 0.0034 to the global difference of these temperature-time curves (see Table 1, column 2). Thus, the weak global difference of these *WT*−mouse lines can now specifically be attributed to a difference in the clearance rates. The *Casp*1*WT* mice offer lower clearance parameter *k* and require noticeably longer time to restore temperature homeostasis compared to other mouse models. This effect is enhanced in *Casp*1*WT*/*Rip*2*KO* when compared to *Casp*1*WT*/*Rip*2*WT* animals. The asymptotically stable normal body temperature *T*_*n*_ and the parameter *r*, related to the force of temperature autoregulation, seems to be genotype independent and have been estimated to be $T_{n}=37.15\;\left(36.87,37.43\right){\;}^{∙}C$ and $r=0.065\;\left(0.049,0.081\right)\;\frac{1}{h}$ (95% CI in parentheses). Since only temperature records up to 24 h are available, an extrapolation of the model prediction beyond this time point has to be done with caution. Assuming an asymptotic temperature *T*_*n*_ is an approximation necessary to avoid an introduction of additional parameters.

**Table 1:** Statistical analysis of pairwise comparisons of temperature-time courses.

	***p*** **-value (NONLIN) / Chisq //** ***p*** **-value ANOVA**				***p*** **-value**	
**Comparison**	**likelihood ratio test**				***t*-test 24*h***	***t*-test AUC**
	**all**	***n* = 6**	***n* = 6**	***n* = 6**	**all**	**all**
*Casp*1*C*284*A* – *Casp*1*C*284*A*/*Rip*2*KO*	<10^−4^/ 71.0	<10^−4^/ 41.9// 0.0002	<10^−4^/ 47.3// 0.0001	<10^−4^/ 47.4// <10^−4^	0.0002	0.0001
*Casp*1*C*284*A* – *Casp*1*KO*	<10^−4^/ 101.5	0.0057/ 13.2// 0.1343	<10^−4^/ 66.3// <10^−4^	<10^−4^/ 38.7// 0.0003	0.0002	<10^−4^
*Casp*1*C*284*A* – *Casp*1*KO*/*Rip*2*KO*	<10^−4^/ 29.5	0.0025/ 15.6// 0.0723	<10^−4^/ 36.5// 0.0008	<10^−4^/ 35.2// 0.0008	0.0135	0.0001
*Casp*1*C*284*A* – *Casp*1*WT*	<10^−4^/ 176.8	0.0098/ 11.9// 0.1343	<10^−4^/ 44.8// 0.0009	0.0003/ 19.4// 0.0338	<10^−4^	0.0001
*Casp*1*C*284*A* – *Casp*1*WT*/*Rip*2*KO*	<10^−4^/ 85.1	0.0052/ 13.6// 0.1343	<10^−4^/ 66.9// <10^−4^	0.0005/ 18.3// 0.0334	<10^−4^	0.0005
*Casp*1*C*284*A*/*Rip*2*KO* – *Casp*1*KO*	0.7042/ 1.4	0.0052/ 13.7// 0.0587	0.3161/ 3.7// 0.5607	0.9257/ 0.5// 0.7943	0.5706	0.7411
*Casp*1*C*284*A*/*Rip*2*KO* – *Casp*1*KO*/*Rip*2*KO*	0.6900/ 1.7	0.0607/ 7.7// 0.1511	0.3861/ 3.0// 0.5723	0.5320/ 2.6// 0.5793	0.8069	0.5764
*Casp*1*C*284*A*/*Rip*2*KO* – *Casp*1*WT*	<10^−4^/ 323.1	<10^−4^/ 75.2// <10^−4^	<10^−4^/ 134.9// <10^−4^	<10^−4^/ 95.0// <10^−4^	<10^−4^	<10^−4^
*Casp*1*C*284*A*/*Rip*2*KO* – *Casp*1*WT*/*Rip*2*KO*	<10^−4^/ 180.6	<10^−4^/ 69.9// <10^−4^	<10^−4^/ 156.8// <10^−4^	<10^−4^/ 102.9// <10^−4^	<10^−4^	<10^−4^
*Casp*1*KO* – *Casp*1*KO*/*Rip*2*KO*	0.3626/ 3.6	0.4429/ 2.9// 0.2736	0.0183/ 10.3// 0.1198	0.9257/ 0.7// 0.5944	0.8575	0.6223
*Casp*1*KO* – *Casp*1*WT*	<10^−4^/ 435.4	<10^−4^/ 32.3// 0.0010	<10^−4^/ 157.1// <10^−4^	<10^−4^/ 83.6// <10^−4^	<10^−4^	<10^−4^
*Casp*1*KO* – *Casp*1*WT*/*Rip*2*KO*	<10^−4^/ 205.2	<10^−4^/ 27.9// 0.0030	<10^−4^/ 176.5// <10^−4^	<10^−4^/ 92.2// <10^−4^	<10^−4^	<10^−4^
*Casp*1*KO*/*Rip*2*KO* – *Casp*1*WT*	<10^−4^/ 176.8	<10^−4^/ 43.4// 0.0001	<10^−4^/ 118.6// <10^−4^	<10^−4^/ 75.7// <10^−4^	<10^−4^	<10^−4^
*Casp*1*KO*/*Rip*2*KO* – *Casp*1*WT*/*Rip*2*KO*	<10^−4^/ 120.4	<10^−4^/ 40.6// 0.0001	<10^−4^/ 148.6// <10^−4^	<10^−4^/ 87.0// <10^−4^	<10^−4^	<10^−4^
*Casp*1*WT* – *Casp*1*WT*/*Rip*2*KO*	0.0034/ 14.1	0.6651/ 1.6// 0.7500	<10^−4^/ 33.1// 0.1932	0.0576/ 8.0// 0.4833	0.3094	0.6986

*Likelihood ratio (LR) tests are either based on nonlinear regression (NONLIN) using model (Equation 5) with pred_ref_ = (T_n_~1, r~genotype, k~genotype, p_0_~genotype) and s = 3, or an ANOVA with interacting independent variables genotype and time. For the LR tests, either the full data set (all) has been used or three reduced data sets with randomly assorted time series (n = 6 per genotype). All effective degrees of freedom for the LR tests are DF = 3. Shown are p-values and the corresponding χ^2^−values [ = 2log(LR)]. Analysis of temperature differences at the time point t = 24h or based on the AUC were performed using two-sided, pairwise t-tests (full data set). All p-values are adjusted for multiple testing using the Benjamini-Hochberg algorithm*.

### 3.3. Total Curve Comparisons of Body Temperature-Time Courses

Comparison of kinetic parameters between experimental groups can allow quantification of biological mechanisms contributing to differences between groups. However, if more than one kinetic parameter strongly depend on the genotype or experimental setting, the overall assessment of experimental groups has to be based on comparisons of entire curves rather than single kinetic parameters. Data resulting from repeated observations or measurements at subsequent time points are often analyzed based on statistical methods that either account for the occurrence of repeated measurements or within-subject dependence. The most important methods are “repeated measures ANOVA,” “generalized estimating equations (GEE),” “Mixed effects model” and “generalized least squares (GLS)” (Harrell, 2001). However, none of these methods allow for a straightforward extension to dynamic models (Harrell, 2001).

Here, two approaches of total curve comparisons are presented. First, a comparative analysis can be performed using the kinetic response model followed by pairwise comparison of experimental groups with measures of differences in goodness-of-fit such as results from likelihood ratio tests (see Table 1, column 2). The second approach consists in choosing an appropriate summary statistic, e.g., area under the curve (AUC) over time, assuming that “the summary measure is an adequate summary of the time profile and assesses the relevant treatment [group] effect” (cf. Klein et al., 2007). The AUC is of particular importance in our case since it serves as a surrogate for an accumulated effect caused by an inflammation-triggered sustained deviation from normal body temperature.

#### 3.3.1. Likelihood-Ratio-Tests Allow to Accurately Distinguish Body Temperature-Time Courses but Disregard Identical Cumulative Measures

The likelihood-ratio-test procedure, as applied here, corresponds to a pairwise comparison of entire curves. The results of all pairwise comparisons (see Table 1) confirmed what we predicted from both: (i) a visual inspection of temperature-time curves (Figure 2) and (ii) the reported genotype-dependent differences in *p*_0_ (see Figure 3). However, LPS stimulation of *Casp*1*WT* and *Casp*1*WT*/*Rip*2*KO* lead to visually rather similar, but temporally shifted temperature-time curves (see Figure 2). Based on a likelihood ratio test, the difference of these two curves is statistically significant (see Table 1). However, the relative effects of inflammation in the two cases are unclear. Therefore, analyzing the area under the curve (AUC) based on the solutions of the mathematical models might be more suitable to reflect the overall strength of inflammation.

#### 3.3.2. Area Under the Curve as an Index of Inflammation

Here, an appropriate definition for the AUC is the area between the line representing the constant normal body temperature, *T*_*n*_ and the predicted temperature curve, *T*(*t*) resulting from LPS stimulation

(6) $$\begin{array}{r} AUC=\overset{t_{max}}{\underset{0}{\int }}T_{n}-T\left(t\right)\;dt. \end{array}$$

The upper limit is chosen to be the final observation time point *t*_*max*_ = 24*h* in the sequel but any value 0 < *t*_*max*_ < ∞ is principally allowed and might be appropriate in other cases since the prediction *T*(*t*) can be extrapolated beyond *t* = *t*_*max*_. In practice, since a closed solution *T*(*t*) is not available, a numeric solution approximating *T*(*t*) with a tiny integration time step is used to numerically compute the AUC based on an approximately smooth fine-stepped linear interpolation. Of note, for a statistical analysis of group differences based on the AUC, it is necessary to compute the prediction curves on the subject level, i.e., for each individual temperature-time course. All *n* = 237 individual fits are shown in the Supplementary Figure 2 along with a plot of the residuals (Supplementary Figure 3). Group-wise differences in AUC can then be tested by means of an ANOVA along with subsequent *post-hoc* pairwise *t*-tests. This leads to (Benjamini-Hochberg adjusted) *p*-values for the pairwise comparisons as listed in Table 1 (last column). Explicitly, group level mean AUCs were calculated as *AUC* = 166.62°*Ch* (sd = 57.94°*Ch*) for *Casp*1*WT* and *AUC* = 161.78°*Ch* (sd = 30.22°*Ch*) for *Casp*1*WT*/*Rip*2*KO* genotypes leading to a non-significant difference (*p* = 0.6986) in contrast to the inference from the likelihood ratio (cf. Table 1). The mutual horizontal shift of the two curves, which does not substantially contribute to the overall damage, is related to a difference in clearance rate *k*, as we have discussed in section 3.2. Worth of note, a calculation of the AUCs for the “averaged” (group level) curves of these two genotypes yields 167.70°*Ch* and 159.26°*Ch*, respectively, fully consistent with the mean AUCs resulting from the individual (subject level) curves.

### 3.4. The Kinetic Response Model Shifts the Focus From Between-Subjects to Within-Subject Variability and Offers Superior Statistical Power

In contrast to the application of pairwise *t*-tests at only one time point, a single temperature-time series from one experimental animal per group may theoretically contain enough information to compare the body temperature between groups based on fitted curves. In other words, like any other repeated measures regression model, the kinetic response model shifts the focus from between-subject to within-subject variability upon increasing the number of repetitions. However, the idealized experiment allowing group-comparisons based on only one mouse per genotype, assumes a sufficiently dense sampling of the temperature-time course and a moderate impact of intra-group variability. The most important practical consequence is obvious and has eminent ethical relevance: Shifting the calculation of a proper sample size needed to significantly distinguish a hypothesized effect with sufficient power, from the necessary number of subjects (here mice) to a sufficient number of within-subject repetitions (here within-mouse temporal sampling frequency). Consequently, an optimal assessment, i.e., a power analysis along with a proper sample size calculation, should be based on a mixed effects model in order to account for possible differences and impacts of the contributing variabilities. However, such an analysis is beyond the scope of this study but the superior statistical power of analyses based on the kinetic response model compared to analyses of single time points can be shown.

Based on the genotypes *Casp*1*KO* (*n*_1_ = 57) and *Casp*1*C*284*A* (*n*_2_ = 64), we used the observed mean temperature difference, Δ*T* = 1.81°*C*, at *t* = 24*h* and the corresponding pooled standard deviation, $SD_{T}=2.03^{∙}C$, to calculate Cohen's effect size as $\Delta_{T}=\frac{\Delta T}{SD_{T}}=0.89$. Assuming a two-sided *t*-test, a power of 80% and a significance level of 0.05 led to a minimal sample size of *n* = 21 mice per group necessary to detect this effect. Applying the same procedure to the AUCs resulting from the kinetic model (ΔAUC = 49.9°*Ch*, $SD_{\text{AUC}}=37.16^{∙}Ch$), we calculated Cohen's effect size as Δ_AUC_ = 1.34. Again assuming a two-sided *t*-test with a power of 80% and a significance level of 0.05, led to a minimal sample size of *n* = 10 mice per group for the AUC-based analysis. To further illustrate the superiority of our biologically motivated kinetic model in comparison to simple *t*-tests, we randomly picked 6 observed time series per experimental group and repeated the pairwise comparison of entire body temperature curves using the likelihood ratio test. Despite using this dramatically reduced sample size we were able to verify the inferences drawn from *t*-tests applied to measurements at *t* = 24*h* or the likelihood ratio tests based on the full sample size (Table 1). For an illustration of the observed time series obtained from two of the three reduced data sets we refer to Supplementary Figure 1.

To compare our kinetic model with a standard statistical method taking all time-points into account, we performed pairwise likelihood ratio tests based on standard linear models (ANOVAs) with genotype and time as predictors including the interaction between these two independent variables. We applied the same three reduced data sets consisting of only 6 time series per experimental group (see Table 1). The ANOVA analysis was able to verify the inferences drawn from *t*-tests applied to the full dataset at *t* = 24*h* for most, but not all reduced datasets. A direct comparison of the ANOVA and the kinetic model applied to these reduced datasets uncovered the ANOVA to be less discriminative. However, since the ANOVA approach assumes a linear dependence on time, whereas in fact the data are clearly very nonlinear in time, it is not surprising that this test would not perform as well as a test from a model that is suited to fit the data curves.

## 4. Discussion

Here, we demonstrate a mathematical modeling approach capable of fitting and analyzing temperature time curves observed in experimental animals. In sub-thermoneutral ambient temperatures, mice respond to LPS with hypothermia, which can be used as surrogate for the severity of systemic inflammation. The used model parameters are kinetically and mechanistically interpretable. In contrast to a purely data-driven approach to analyze the observed curves, the mathematical modeling approach offers the ability to compare entire body temperature curves on a deterministic basis and adds additional information by introducing the interpretable parameters normal body temperature (*T*_*n*_), a coefficient related to the force of temperature autoregulation (*r*), damage strength (*p*_0_) and clearance rate (*k*). Therefore, the kinetic model is also a theoretical result (Goldstein, 2018).

The normal body temperature *T*_*n*_ and the parameter *r*, related to the force of thermal autoregulation, showed no dependence on the genotypes analyzed. Both parameters together affect the minimum body temperature and the temporal progression of the recovery. For the data analyzed in this study, differences between groups depend mainly on the damage strength *p*_0_. However, the difference between *Casp*1*WT* and *Casp*1*WT*/*Rip*2*KO* solely depends on a difference in clearance parameter *k*. The onset and the clearance of inflammation is mediated through a complex network of cells and pro- and anti-inflammatory mediators, but the phenomenological mathematical model presented here subsumes pro-inflammatory mechanisms mainly reflected by damage strength *p*_0_ and anti-inflammatory mechanisms mainly as clearance rate *k*. Therefore, detailed biological interpretation on the cellular and molecular level of these parameters is not feasible, whereas it allows an approximate estimation of both mechanisms contributing to the inflammatory response. This is consistent with hypothermia conceived as surrogate for systemic inflammation because hypothermia also represents a sum signal of pro- and anti-inflammatory mechanisms.

Although differences in LPS-induced inflammation are mainly introduced by genotypes (*Casp*1*WT* > *Casp*1*C*284*A* > *Casp*1*KO*), differences can be found also within experimental groups: Nearly all animals began to restore their body temperature within 24 h of observation, but some animals did not (see Supplementary Figure 2). For these animals, the host's limit of tolerable systemic inflammation might be exceeded, and the animals are suspected to die subsequently. We believe that these differences in systemic inflammation are based on interindividual variability.

The mathematical model is able to sufficiently predict the temperature-time curve of the experimental animals, and therefore it is applicable to compare overall systemic inflammation between experimental groups as well. Pairwise comparisons of modeled temperature-time curves can be performed using likelihood-ratio tests or the AUC as a summary measure. Although some pairs of modeled temperature-time curves may exhibit statistically significant differences when analyzed using a likelihood-ratio test, these differences might be caused rather by temporal delays of the curves (*Casp*1*WT* vs. *Casp*1*WT*/*Rip*2*KO* in our data set) than by differences in the severity of systemic inflammation. Therefore, it is important to define whether the temporal course of inflammation or the overall inflammation is of main interest. The likelihood-ratio test sensitively detects differences in the temporal course, whereas the AUC-based tests detect differences in overall systemic inflammation only. Hence, AUCs are insensitive for detecting differences in kinetic clearance and regulation parameters *k* and *r*. If a temporal delay of the dynamics becomes important, e.g., for the estimation of appropriate time points for interventions, the explicit group-dependent evaluations of kinetic parameters turned out to be superior. In this case, goodness-of-fit of the dynamical model offers an adequate basis for comparisons. When interested in overall systemic inflammation mainly, performing likelihood ratio tests for group comparisons as discussed above while keeping the parameters *T*_*n*_, *r* and *k* group-independent (i.e., constant over all experimental groups), and estimating only *p*_0_ as group-specific, is a further option. Of note, the reduction of degrees of freedom also reduces general test power (Makin and Orban de Xivry, 2019). Thus, a knowledge-driven (not merely dominated by the *p*-value or by the power) reduction of degrees of freedom allows to focus on biologically important parameters while keeping the power to detect relevant effects related to these parameters as high as possible.

Analyzing the AUC typically requires a sufficient duration of temperature measurements until approximately returning to the normal body temperature. The kinetic modeling approach allows for inter- and extrapolations, respectively. Therefore, it is legitimate to follow-up the time courses beyond the final observation time to observe the asymptotic process of stabilizing the temperature back to its normal body temperature. However, such an extrapolation may increase the coefficient of variation of the AUCs. Applying the AUC analysis to our data set using *t*_*max*_ = 24*h*, corresponding to the terminal observation time of the experimental measurements, turns out to be a good strategy in estimating the effective systemic inflammation as basis for comparisons of the experimental groups. In principle, the AUC could be calculated based on the actual observations when using a linear or more complex interpolation as, e.g., a cubic spline. Due to the large temporal gaps between the observations in our data set, a linear interpolation is obviously not advantageous. A spline approximation is smooth but incompatible with any biological interpretation.

Experimental animals in LPS shock models suffer from systemic inflammation. Whenever possible, the 3R principle (Replacement, Reduction, Refinement) should be addressed in order to improve the animal welfare. Using hypothermia instead of animal death as endpoint and applying mathematical modeling of the resulting time temperature data strongly applies to the 3R principle. First, using hypothermia reduces animals suffering from death (Refinement) and additionally allows the analysis of recovery from inflammation as well as measuring serum cytokines at specific time points from the same animals (Reduction). Second, the mathematical-statistical approach presented in this study shifts the source for its power from sample size of experimental groups to within-subject repeated measurements and provides enhanced overall-statistical power when compared to the analysis of single time points using *t*-tests or ANOVA analysis. Therefore, a strong reduction in the required group size can be achieved (Reduction). Moreover, the essential disadvantage of single time point analyses can be resolved: The difference between two group-specific curves substantially differing in the magnitude of minimum temperature but converging at time *t* = 24*h* is detected when using the modeling approach, whereas it is missed when analyzing the curves at *t* = 24*h* only. In this context, using the entire curves or the AUCs as integral measures of the curves to compare groups, implies a different conception of effect size in comparison with the analysis of a single time point. Unique mutual transformations of these effect sizes do not exist.

From the epistemological point of view, it has been emphasized as early as 1961 by Turner et al. (1961) that “the biometrician must be concerned not only with the efficiency of his estimation procedures but also with the adequacy of his descriptive model. […] In some cases it is possible to derive rather sophisticated theoretical models on the basis of acquired knowledge and intelligent hypothesizing. These models are often conveniently found as solutions of differential equations.” Only if nothing is known about the mechanisms that drive the observed dynamics “one may resort to polynomial models […]” or a simple repeated-measures analysis of (co-)variance. The strength of the mathematical model presented here is the capability to capture the mechanistic basis of the response kinetics on a surrogate level by means of a biologically interpretable dynamical model rather than purely statistical improvements of an arbitrary non-linear regression. Thus, the theory-driven approach offers a better understanding of involved biological processes via the evaluation of the kinetic parameters rather than just detecting differences that do not allow proper interpretation on the basis of mainly data-driven approaches.

The kinetic response model presented in this study was tested using LPS stimulated C57BL/6N mice. However, as different mouse strains and rats share similar hypothermic responses to LPS (Vlach et al., 2000; Dogan et al., 2002; Steiner et al., 2011), the model might be applicable to analyze these experimental animals as well. Models of sterile endotoxemia like the intraperitoneal LPS model typically lead to acute temporary inflammation due to the rapid rise of the endotoxin concentration and its short half-life in the circulation. Interestingly, systemic application of TNFα leads to hypothermic responses comparable to those found after LPS stimulation (Cauwels et al., 2009; Huys et al., 2009). Therefore, the mathematical model might be reliably equipped to analyze body temperature curves following systemic application of other sterile PAMPs or DAMPs as well. Furthermore, temperature-time curves obtained from murine models of bacterial septic or anaphylactic shock are also comparable to those obtained from sterile endotoxin shock (Hox et al., 2016; Li et al., 2018; Vandewalle et al., 2019). However, shock models using viable bacteria might additionally lead to prolonged inflammation and late death of animals despite regaining normal body temperature. Therefore, the kinetic model proposed here may be applicable only for the quantification of the early acute inflammatory response unless the set of kinetic parameters is extended in order to account for mortality or other state transitions. Anyhow, the application of the proposed mathematical model might require optimization of the distinct shock protocol in order to avoid early animal death for all experimental groups. Besides hypothermia, febrile responses can be induced in mouse models as well. In order to induce fever, lower LPS doses and thermoneutral ambient temperatures are needed (Rudaya et al., 2005). The mathematical model was not adapted for febrile responses. As the modeling idea—aberration from a normal temperature—fits to the febrile response as well, changing to negative *p*_0_ may allow a straightforward adaption of the kinetic model.

Some limitations of the study should be noted. Firstly, lacking knowledge about mathematical modeling might prevent researchers from using this approach. However, the complexity of our modeling approach is comparable to pharmaco-kinetic and dose-response models, which are used for the quantitative assessment of clearance dynamics and receptor-ligand affinities. For example, a dose-response analysis tool comparable to the *drc*-package introduced in Ritz et al. (2015) is suitable to foster standardized and good-practice applications of our modeling framework. Secondly, a rigorous power analysis based on a statistical mixed-effects model, which adequately considers the different involved random effects and allows to identify an optimal balance between within-subject repeated measurement and the number of subjects remains to be done. Unfortunately, the implementation of mixed-effect modeling in statistical software in combination with dynamical modeling has not yet found an acceptable standard. The available mixed-effects version *medrc* of the *drc*-package is not able to include differential equation based models (Gerhard and Ritz, 2017) but is a step in the right direction. Thirdly, we tested in an explorative way whether a less complex homeostatic control in form of *dT*/*dt* = *r*(*T*_*n*_−*T*) will suffice. This form has been previously used (Hammel et al., 1963) and is often referred to as “proportional control.” Although this simpler regulation performs well in many cases, we opted for the nonlinear logistic regulation due to its flexibility. Moreover, it has been recently shown that the classical linear approach is over-simplified and a nonlinear form is needed (Boldrini et al., 2018). Close to the equilibrium, the simpler model corresponds to a Taylor approximation of the nonlinear model. However, we did not test robustness of our model against other models of similar complexity, which remains to be investigated. Finally, experimental animals were not systematically habituated to handling procedures. It is known that stressful procedures like intraperitoneal injections and temperature measurements using rectal probes can confound body temperature data and block or induce early febrile responses (Kozak et al., 1994; Rudaya et al., 2005). Since temperature data was obtained until *t* = 24*h* only, we cannot exclude late febrile responses as well. Therefore, the LPS protocol used for this study does not represent the best practice approach and may introduce at least some experimental bias. However, we aimed for the analysis of the main hypothermic response which is known to represent a surrogate of systemic inflammation and treated all experimental groups identically. The LPS stimulation—as performed in our study—is widely used in the scientific literature and we were able to detect differences in hypothermic responses between experimental groups using this approach (Ochalski et al., 1993; Saito et al., 2003; Nold et al., 2010; Cauwels et al., 2013; Berghe et al., 2014; Vandendriessche et al., 2014; Mei et al., 2018; Reinke et al., 2020). We believe that this LPS protocol is suitable to analyze hypothermic response curves and hence, systemic inflammation in mice. Therefore, the mathematical model fitted to temperature-time series resulting from this approach provides an additional analysis strategy to the scientific field.

Taken together, knowledge-driven kinetic modeling combined with data-driven statistical modeling offers the possibility to increase the accuracy of assessing body temperature-time courses and to substantially reduce the minimum number of experimental animals needed to reveal relevant effects in the context of experiments on pro-inflammatory signaling. The kinetic model is composed of biologically interpretable components and allows to estimate onset and clearance of inflammation in whole organisms. Using likelihood ratio- or AUC-based analyses, the model supports the sensitive analysis of inter-group differences in the temporal course or overall inflammation. The proposed dynamic model has moderate complexity and might be used as a standard method for assessing hypothermic response curves.

## Data Availability Statement

The datasets generated for this study can be found in online repositories. The names of the repository/repositories and accession number(s) can be found below: https://github.com/Diebner/LPS-Shock.

## Ethics Statement

The animal study was reviewed and approved by Landesdirektion Sachsen, Dresden, Germany.

## Author Contributions

HD and SW conceptualized the project. SR performed the experiments. HD and SW analyzed the data. HD performed mathematical modeling. HD, SR, AR-W, and SW wrote the manuscript. All authors contributed to the article and approved the submitted version.

## Conflict of Interest

The authors declare that the research was conducted in the absence of any commercial or financial relationships that could be construed as a potential conflict of interest.

## Publisher's Note

All claims expressed in this article are solely those of the authors and do not necessarily represent those of their affiliated organizations, or those of the publisher, the editors and the reviewers. Any product that may be evaluated in this article, or claim that may be made by its manufacturer, is not guaranteed or endorsed by the publisher.
