# Supplementary material for: A Kinetic Response Model for Standardized Regression Analyses of Inflammation-Triggered Hypothermic Body Temperature-Time Courses in Mice
Source: Front Physiol. 2021 Aug 24;12:634510. doi: 10.3389/fphys.2021.634510 (PMC8421519; doi:10.3389/fphys.2021.634510)
Supplement: Supplementary file 1 [file Data_Sheet_1.PDF]

Supplement to:

Hans H. Diebner, Sören Reinke, Angela Rösen-Wolff, and Stefan Winkler

A kinetic response model for standardized regression analyses of inflammation-triggered hypothermic body temperature-time courses in mice

Fig. S1: Predicted temperature-time courses corresponding to fig. 2, resulting from a fit to the full data set contrasted with six individual observed temperature-time courses per genotype. Panels A-C show a set of six observed time series per genotype which exactly corresponds to one of the three reduced data sets used for demonstrating higher power in section 3.4. A second reduced data set with 6 time series per genotype is depicted in panels D-F.

Fig. S2: Model fits to all n=237 individual observed temperature-time courses T(t) using least squares applied to logarithmized temperature values. Shown are the model predictions along with the observed data points of one experimental animal. For the fits, the same model was used as in fig. 2 (model eq. 5 with s=3). Units: Temperature, T, in °C and time, t, in hours.

Fig. S3: Standardized residuals for all 5 time points of n=237 model fits to the individual observed temperature-time courses. Least squares fits have been applied to logarithmized temperature values. For the fits, the same model was used as in fig. 2 (model eq. 5 with s=3).

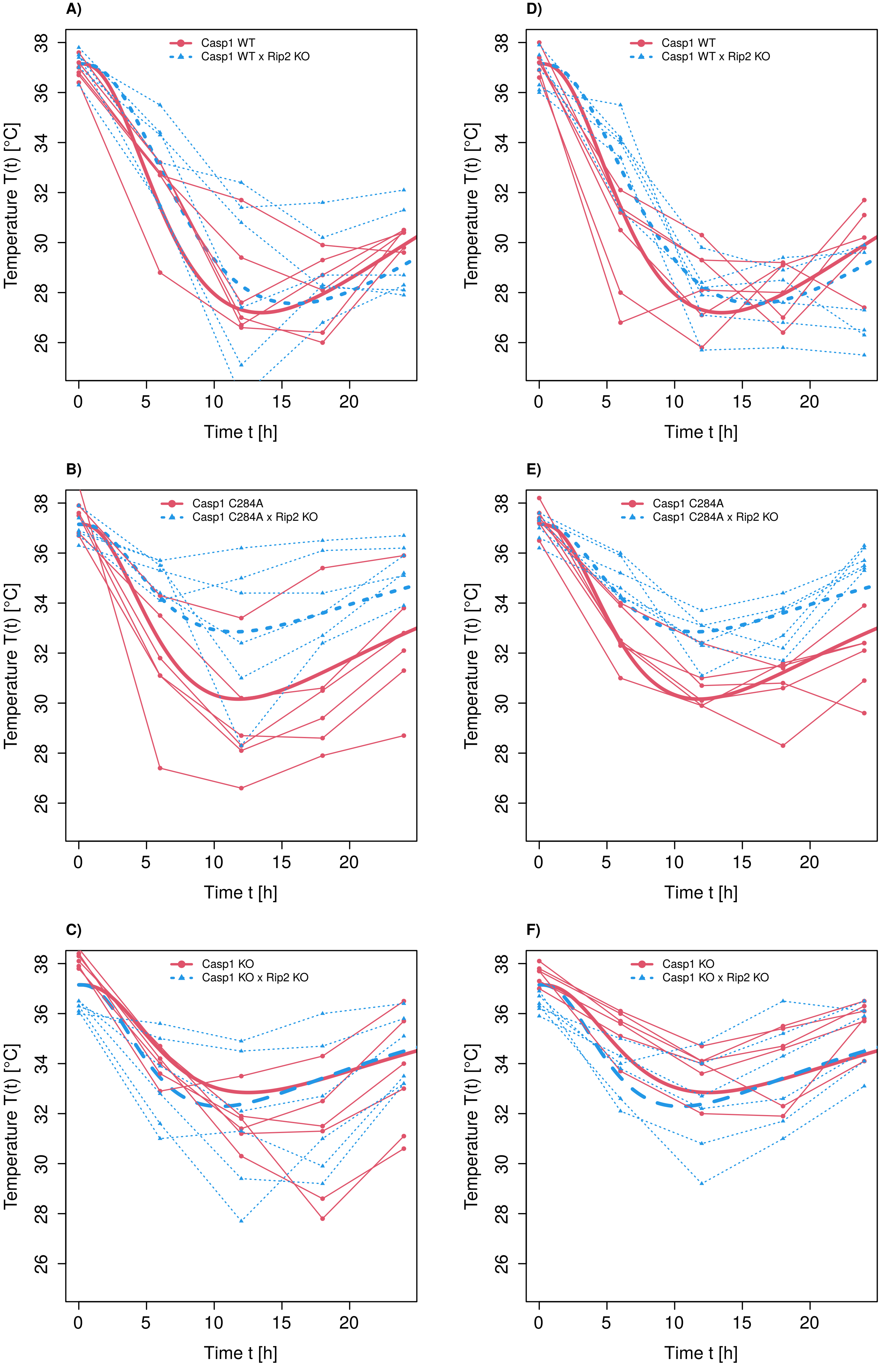
A)
Casp1 WT
Casp1 WT x Rip2 KO
B)
Casp1 C284A
Casp1 C284A x Rip2 KO
C)
Casp1 KO
Casp1 KO x Rip2 KO
D)
Casp1 WT
Casp1 WT x Rip2 KO
E)
Casp1 C284A
Casp1 C284A x Rip2 KO
F)
Casp1 KO
Casp1 KO x Rip2 KO
Temperature T(t) [°C]
Time t [h]
26
28
30
32
34
36
38
0
5
10
15
20

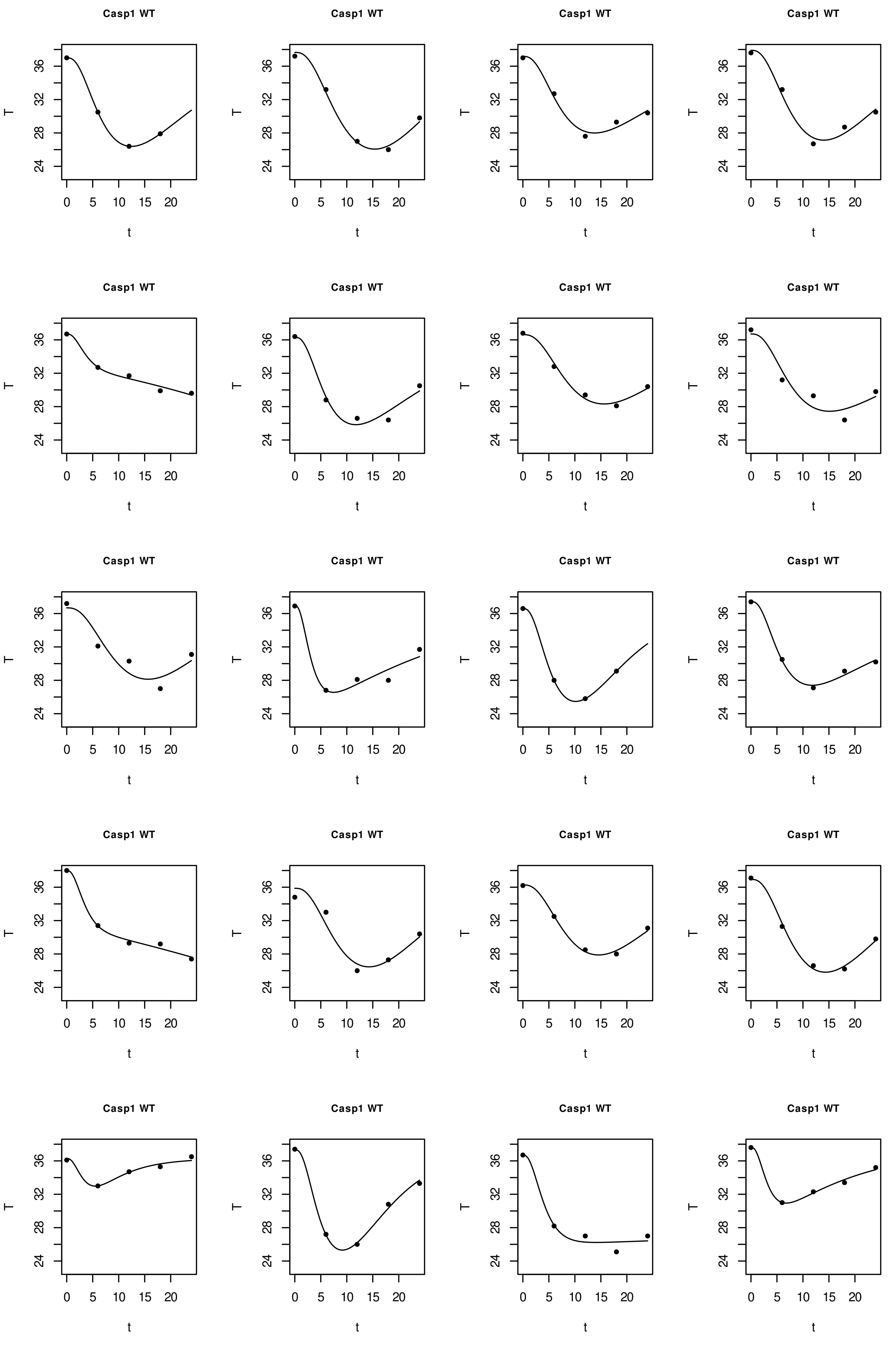
Casp1 WT
Casp1 WT
Casp1 WT
Casp1 WT
Casp1 WT
Casp1 WT
Casp1 WT
Casp1 WT
Casp1 WT
Casp1 WT
Casp1 WT
Casp1 WT
Casp1 WT
Casp1 WT
Casp1 WT
Casp1 WT
Casp1 WT
Casp1 WT
Casp1 WT
Casp1 WT
T
t
24
28
32
36
0
5
10
15
20

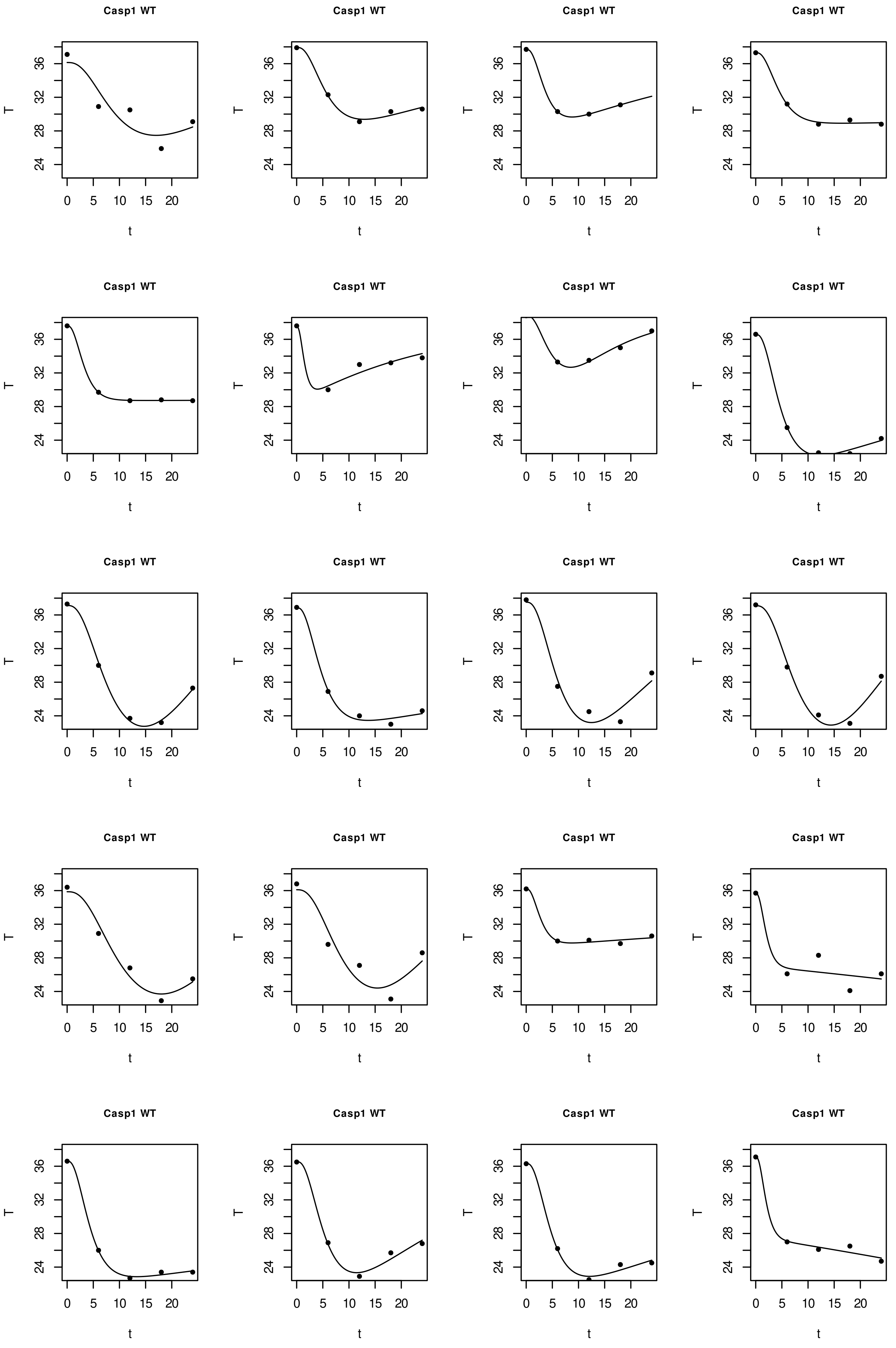
Casp1 WT
T
t
24
28
32
36
0
5
10
15
20

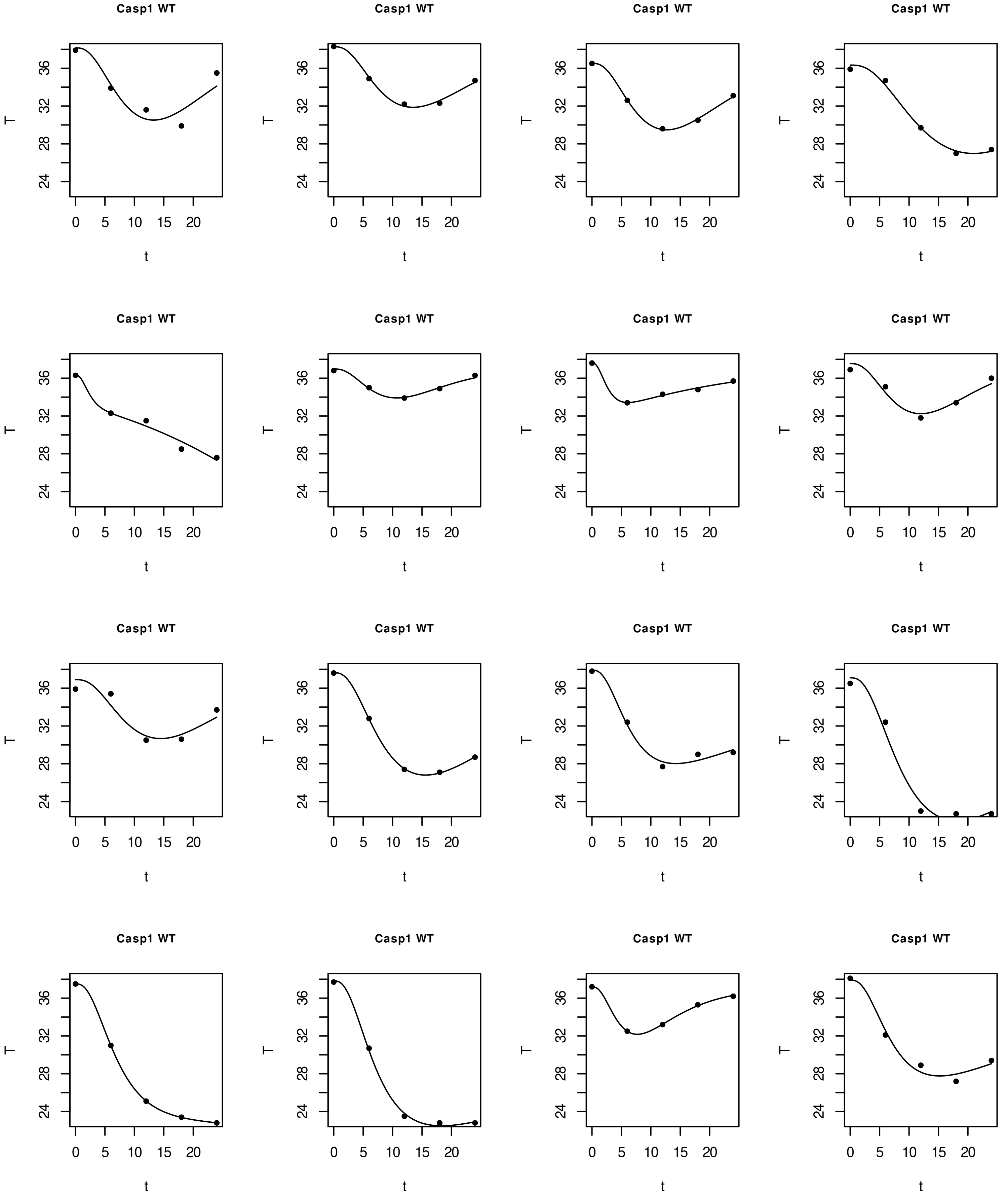
Casp1 WT
Casp1 WT
Casp1 WT
Casp1 WT
Casp1 WT
Casp1 WT
Casp1 WT
Casp1 WT
Casp1 WT
Casp1 WT
Casp1 WT
Casp1 WT
Casp1 WT
Casp1 WT
Casp1 WT
Casp1 WT
T
24
28
32
36
t
0
5
10
15
20

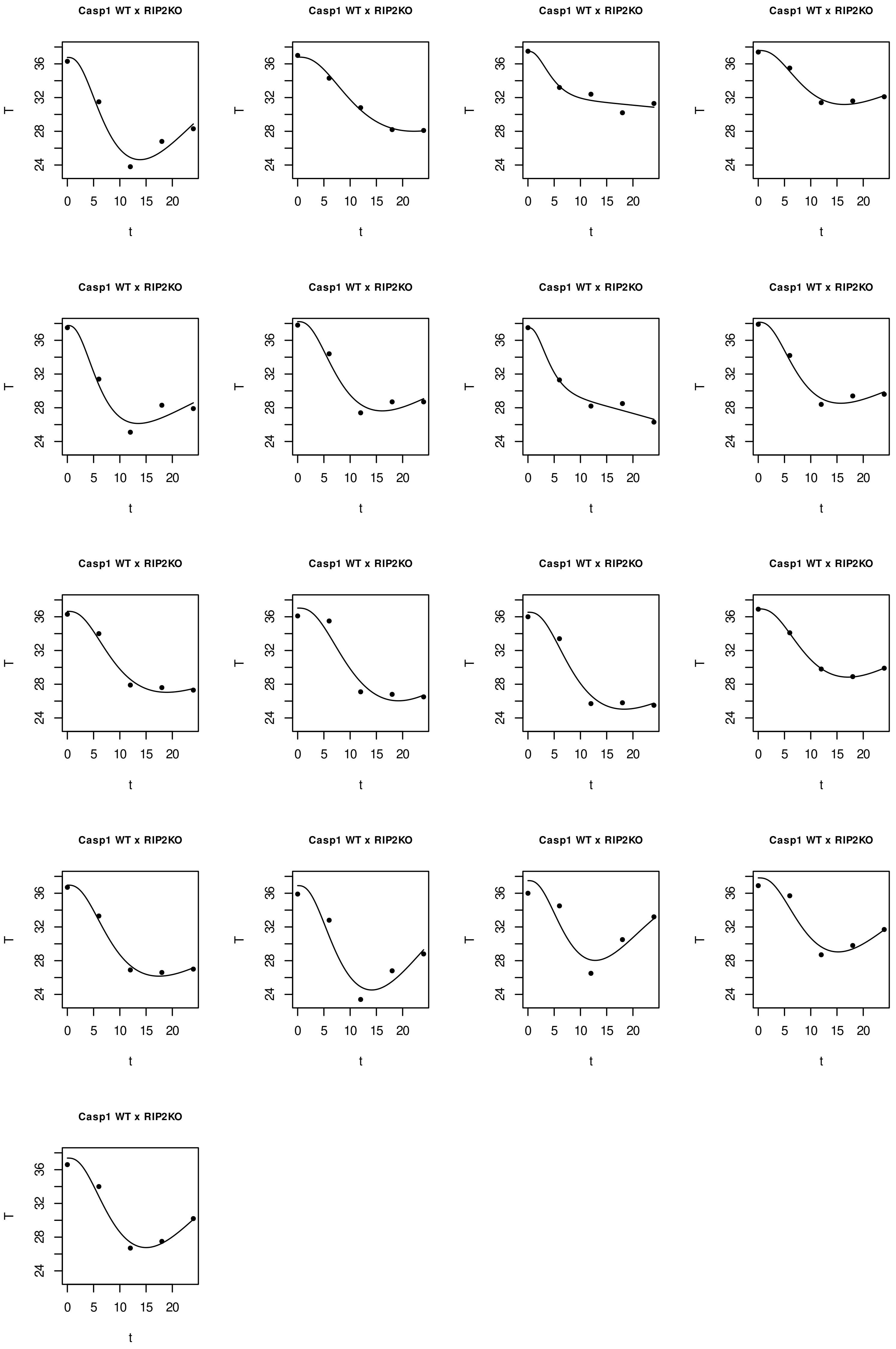
Casp1 WT x RIP2KO
Casp1 WT x RIP2KO
Casp1 WT x RIP2KO
Casp1 WT x RIP2KO
Casp1 WT x RIP2KO
Casp1 WT x RIP2KO
Casp1 WT x RIP2KO
Casp1 WT x RIP2KO
Casp1 WT x RIP2KO
Casp1 WT x RIP2KO
Casp1 WT x RIP2KO
Casp1 WT x RIP2KO
Casp1 WT x RIP2KO
Casp1 WT x RIP2KO
Casp1 WT x RIP2KO
Casp1 WT x RIP2KO
Casp1 WT x RIP2KO
T
t
24
28
32
36
0
5
10
15
20

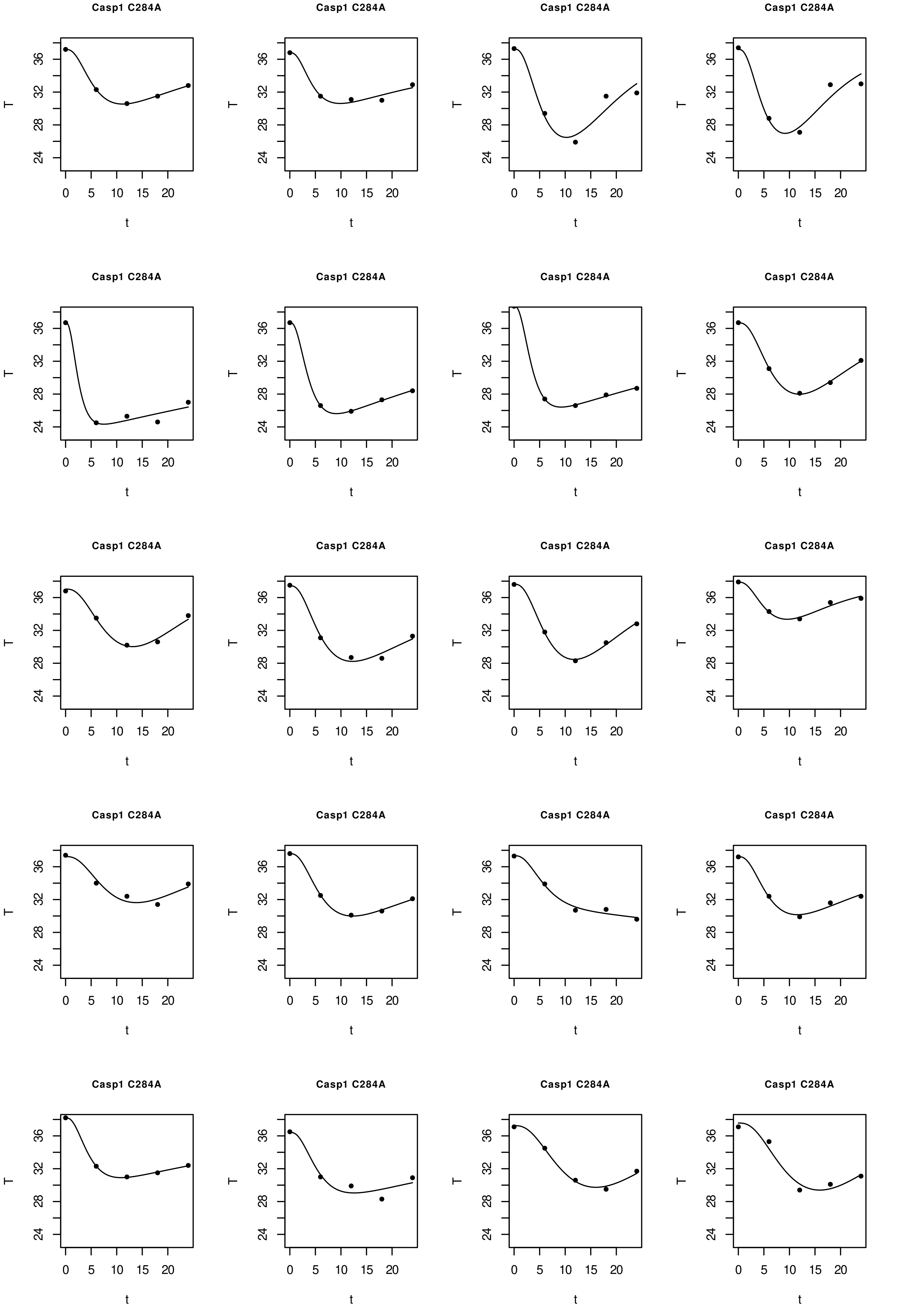
Casp1 C284A
Casp1 C284A
Casp1 C284A
Casp1 C284A
Casp1 C284A
Casp1 C284A
Casp1 C284A
Casp1 C284A
Casp1 C284A
Casp1 C284A
Casp1 C284A
Casp1 C284A
Casp1 C284A
Casp1 C284A
Casp1 C284A
Casp1 C284A
Casp1 C284A
Casp1 C284A
Casp1 C284A
Casp1 C284A
T
t
24
28
32
36
0
5
10
15
20

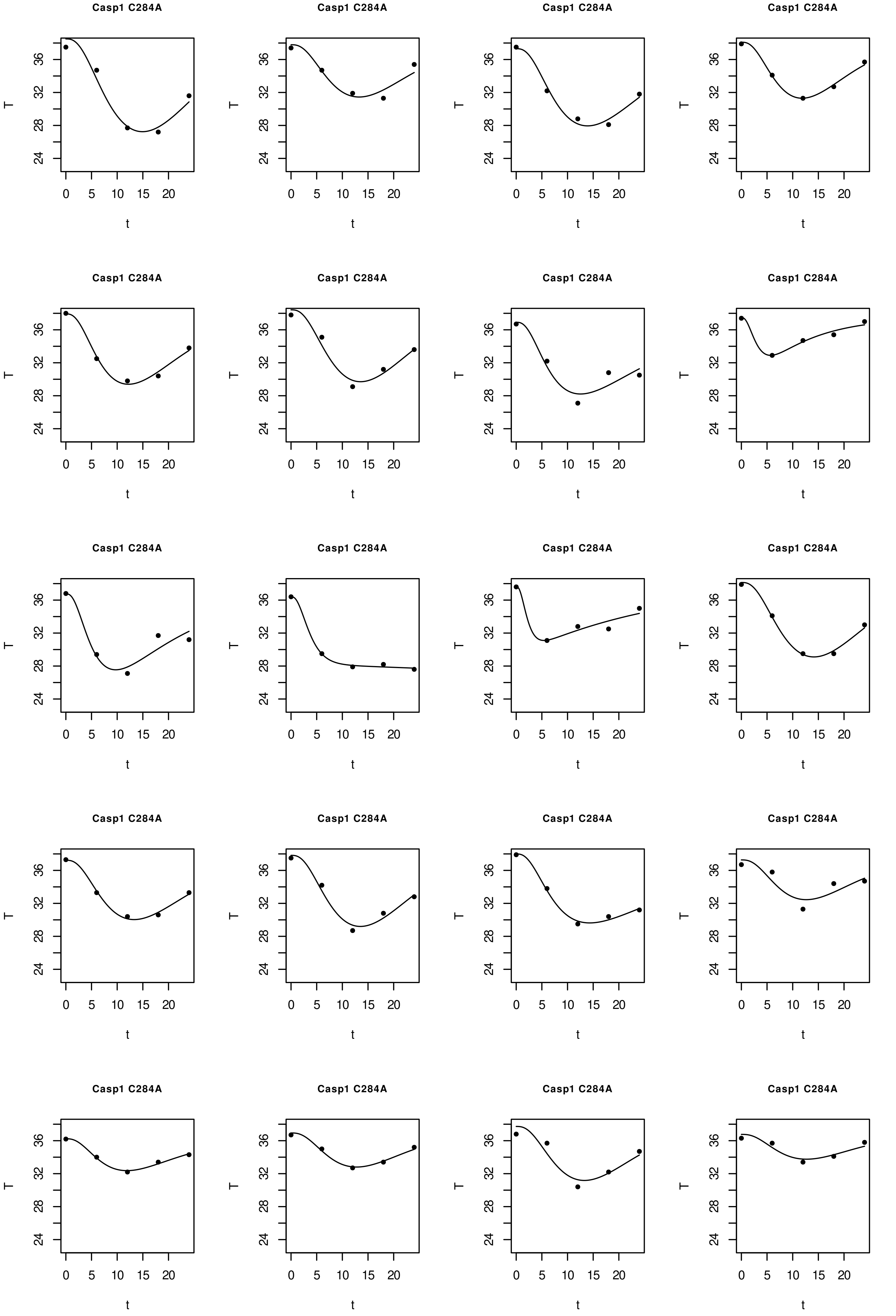
Casp1 C284A
Casp1 C284A
Casp1 C284A
Casp1 C284A
Casp1 C284A
Casp1 C284A
Casp1 C284A
Casp1 C284A
Casp1 C284A
Casp1 C284A
Casp1 C284A
Casp1 C284A
Casp1 C284A
Casp1 C284A
Casp1 C284A
Casp1 C284A
Casp1 C284A
Casp1 C284A
Casp1 C284A
Casp1 C284A
T
24
28
32
36
t
0
5
10
15
20

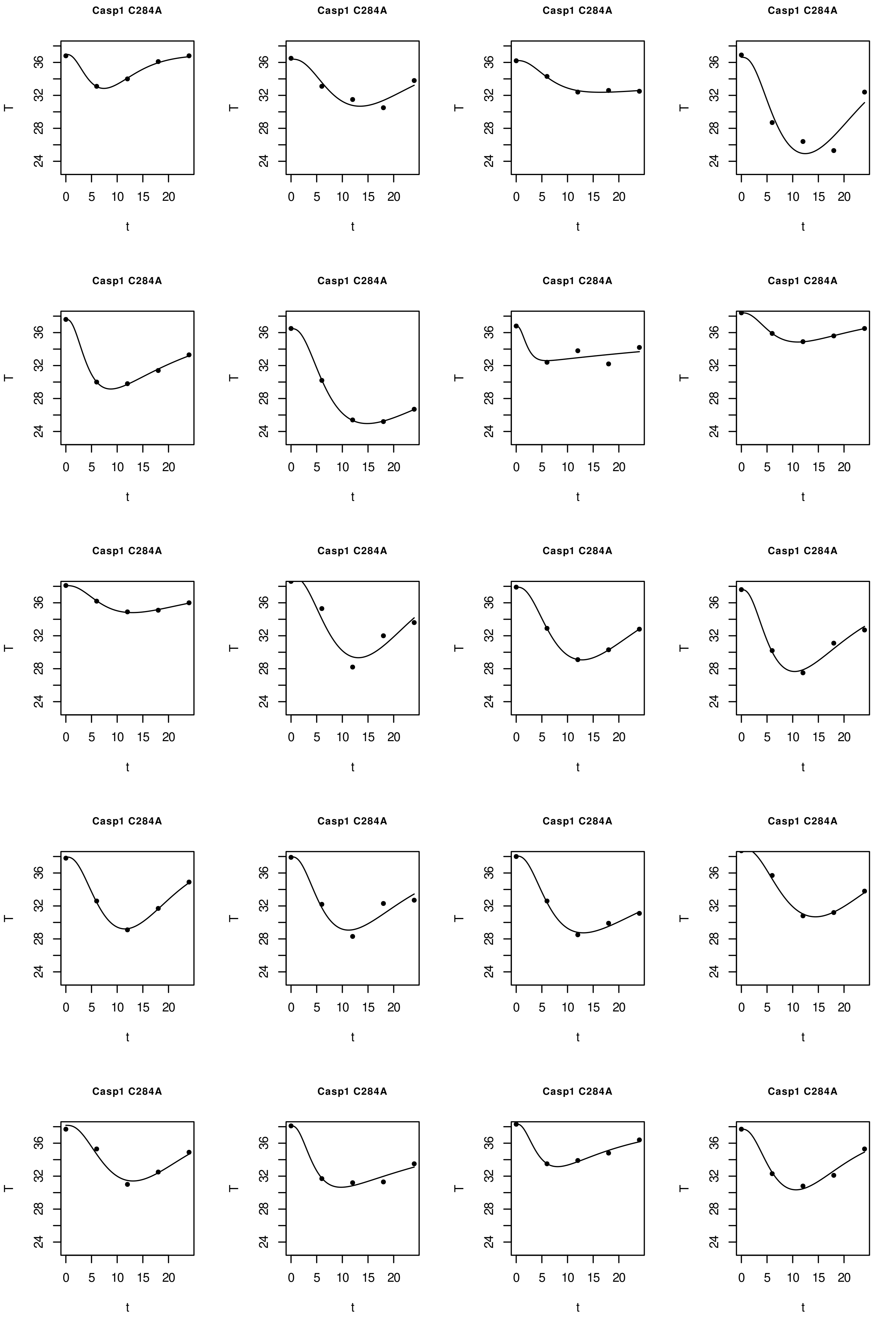
Casp1 C284A
Casp1 C284A
Casp1 C284A
Casp1 C284A
Casp1 C284A
Casp1 C284A
Casp1 C284A
Casp1 C284A
Casp1 C284A
Casp1 C284A
Casp1 C284A
Casp1 C284A
Casp1 C284A
Casp1 C284A
Casp1 C284A
Casp1 C284A
Casp1 C284A
Casp1 C284A
Casp1 C284A
Casp1 C284A
T
t
24
28
32
36
0
5
10
15
20

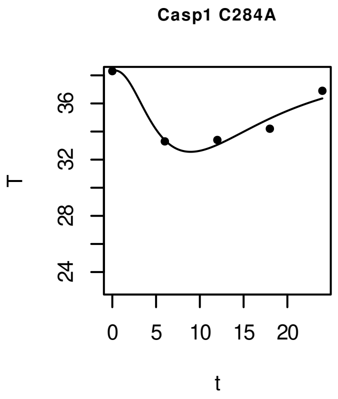
Casp1 C284A
T
36
32
28
24
0
5
10
15
20
t
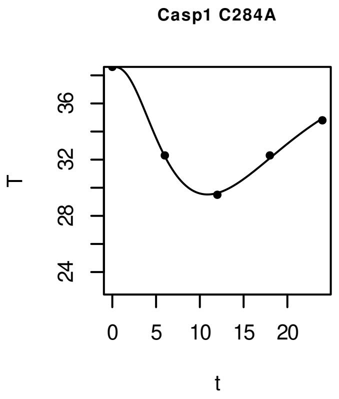
Casp1 C284A
T
36
32
28
24
0
5
10
15
20
t
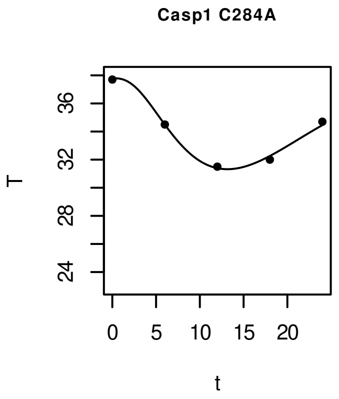
Casp1 C284A
T
36
32
28
24
0
5
10
15
20
t
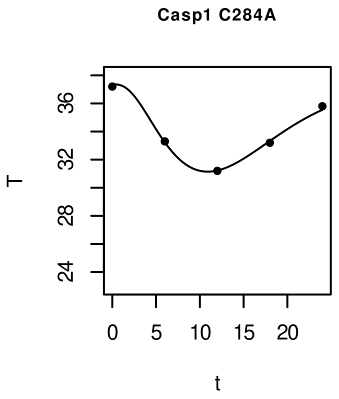
Casp1 C284A
T
36
32
28
24
0
5
10
15
20
t

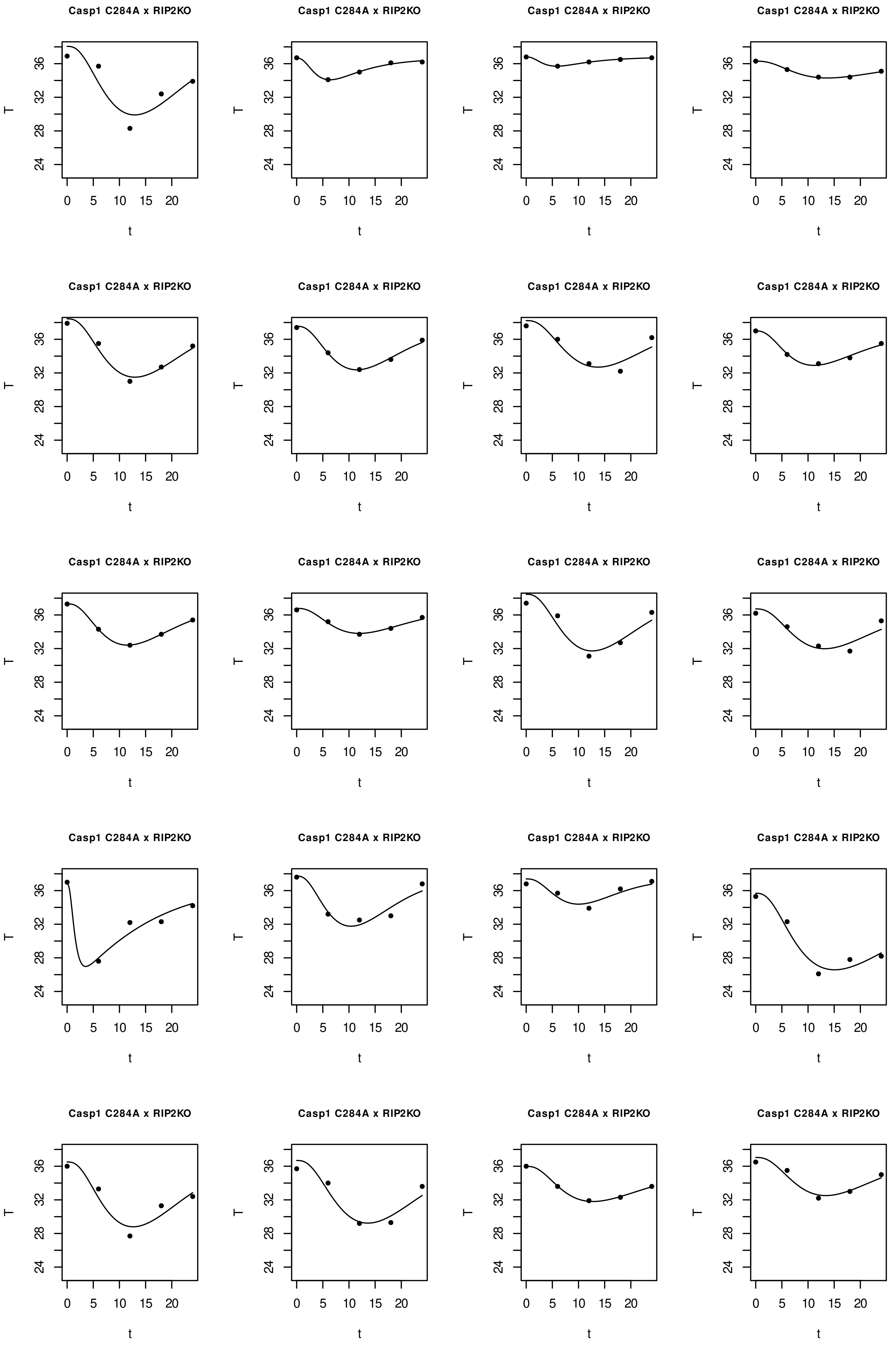

Casp1 C284A x RIP2KO
T
t
24
28
32
36
0
5
10
15
20

Casp1 C284A x RIP2KO

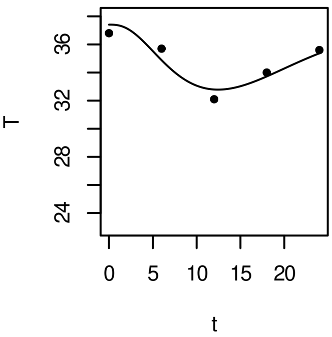

Casp1 C284A x RIP2KO

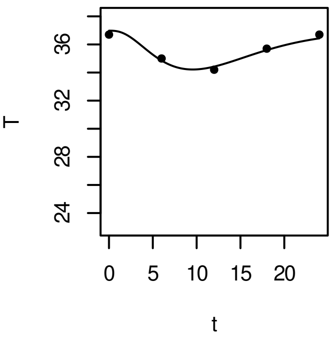

Casp1 C284A x RIP2KO

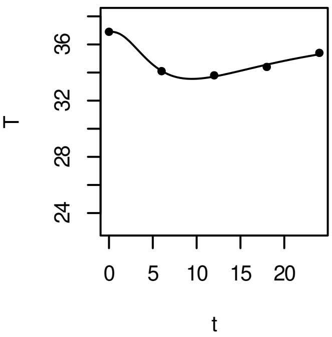

Casp1 C284A x RIP2KO

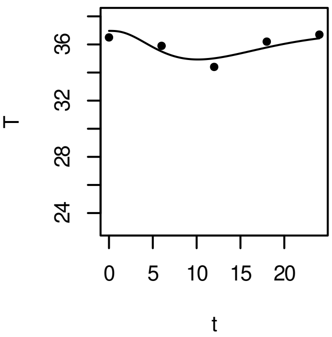

Casp1 C284A x RIP2KO

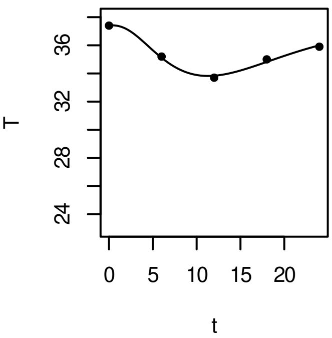

Casp1 C284A x RIP2KO

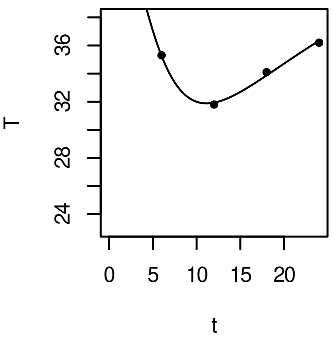

Casp1 C284A x RIP2KO

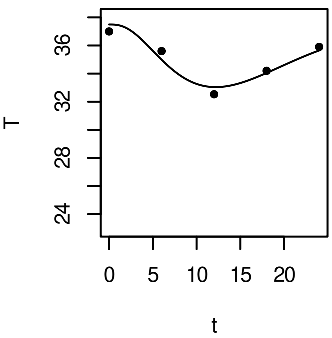

Casp1 C284A x RIP2KO

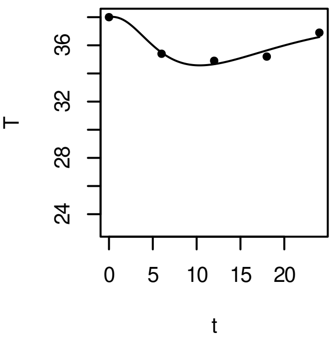

Casp1 C284A x RIP2KO

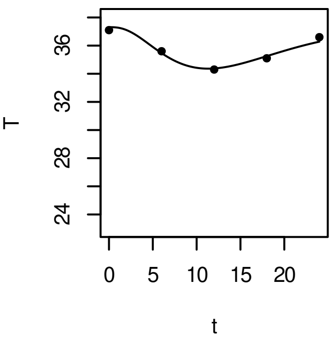

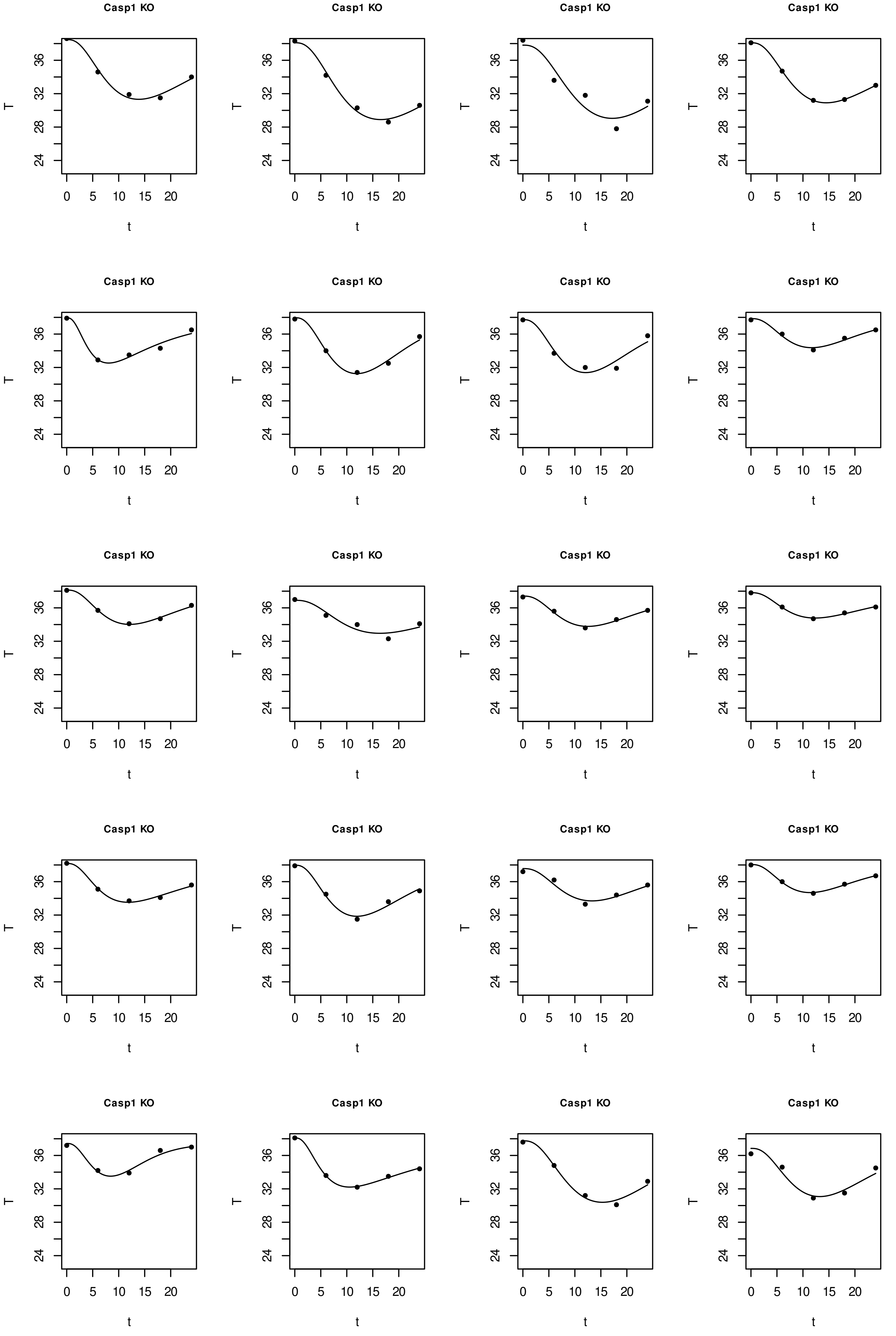
Casp1 KO
Casp1 KO
Casp1 KO
Casp1 KO
Casp1 KO
Casp1 KO
Casp1 KO
Casp1 KO
Casp1 KO
Casp1 KO
Casp1 KO
Casp1 KO
Casp1 KO
Casp1 KO
Casp1 KO
Casp1 KO
Casp1 KO
Casp1 KO
Casp1 KO
Casp1 KO
T
t
24
28
32
36
0
5
10
15
20

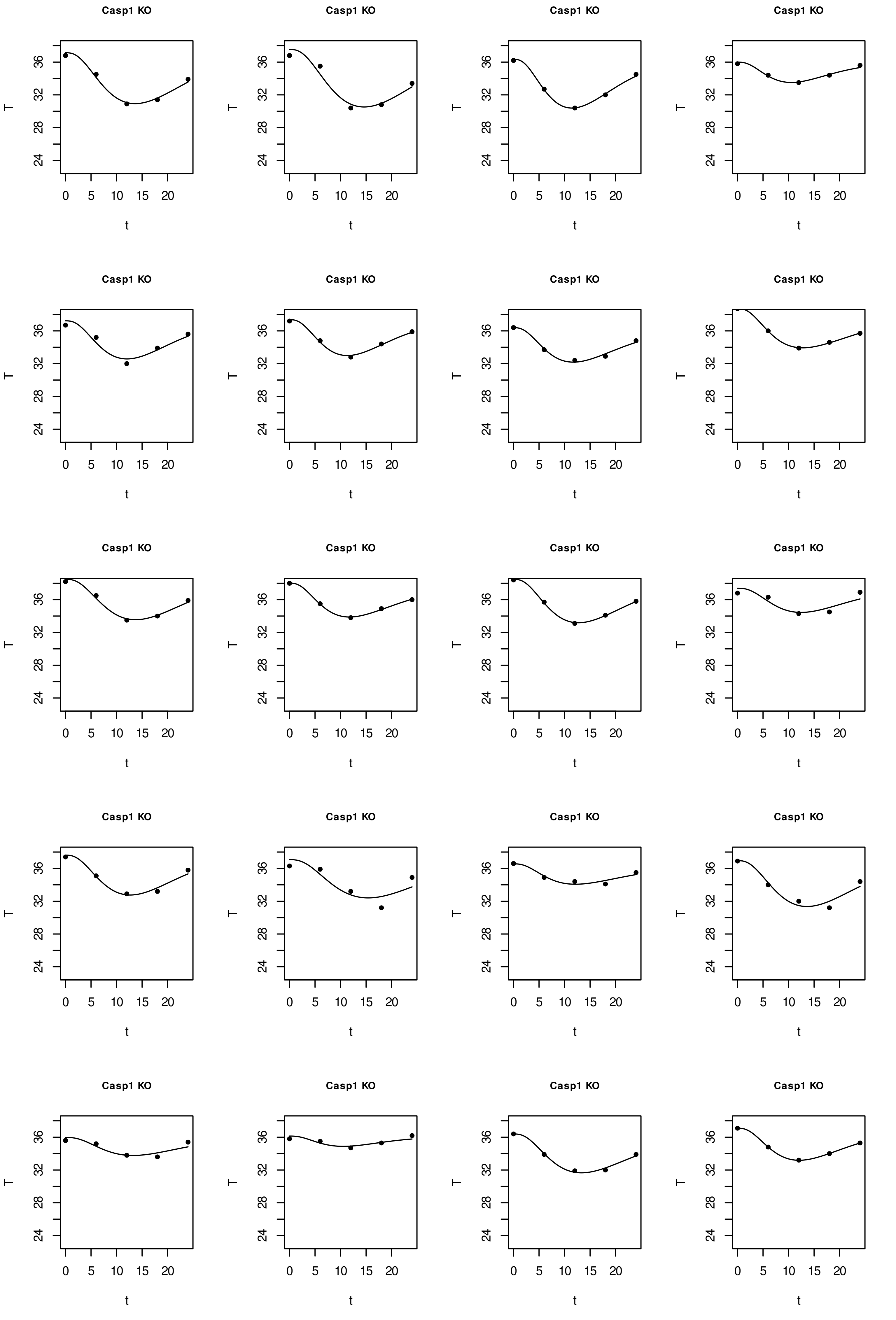
Casp1 KO
Casp1 KO
Casp1 KO
Casp1 KO
Casp1 KO
Casp1 KO
Casp1 KO
Casp1 KO
Casp1 KO
Casp1 KO
Casp1 KO
Casp1 KO
Casp1 KO
Casp1 KO
Casp1 KO
Casp1 KO
Casp1 KO
Casp1 KO
Casp1 KO
Casp1 KO
T
24
28
32
36
t
0
5
10
15
20

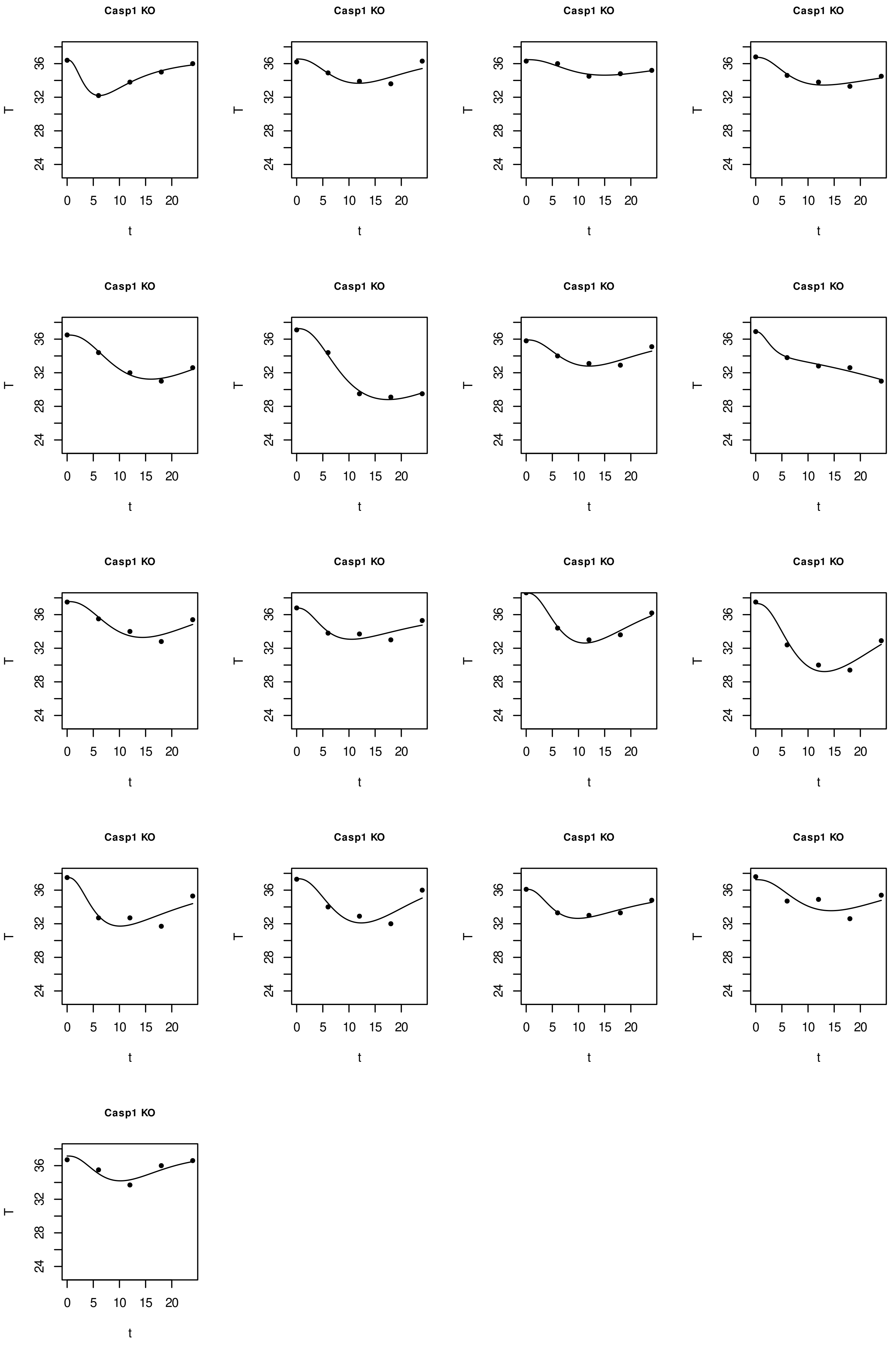
Casp1 KO
T
t
24
28
32
36
0
5
10
15
20

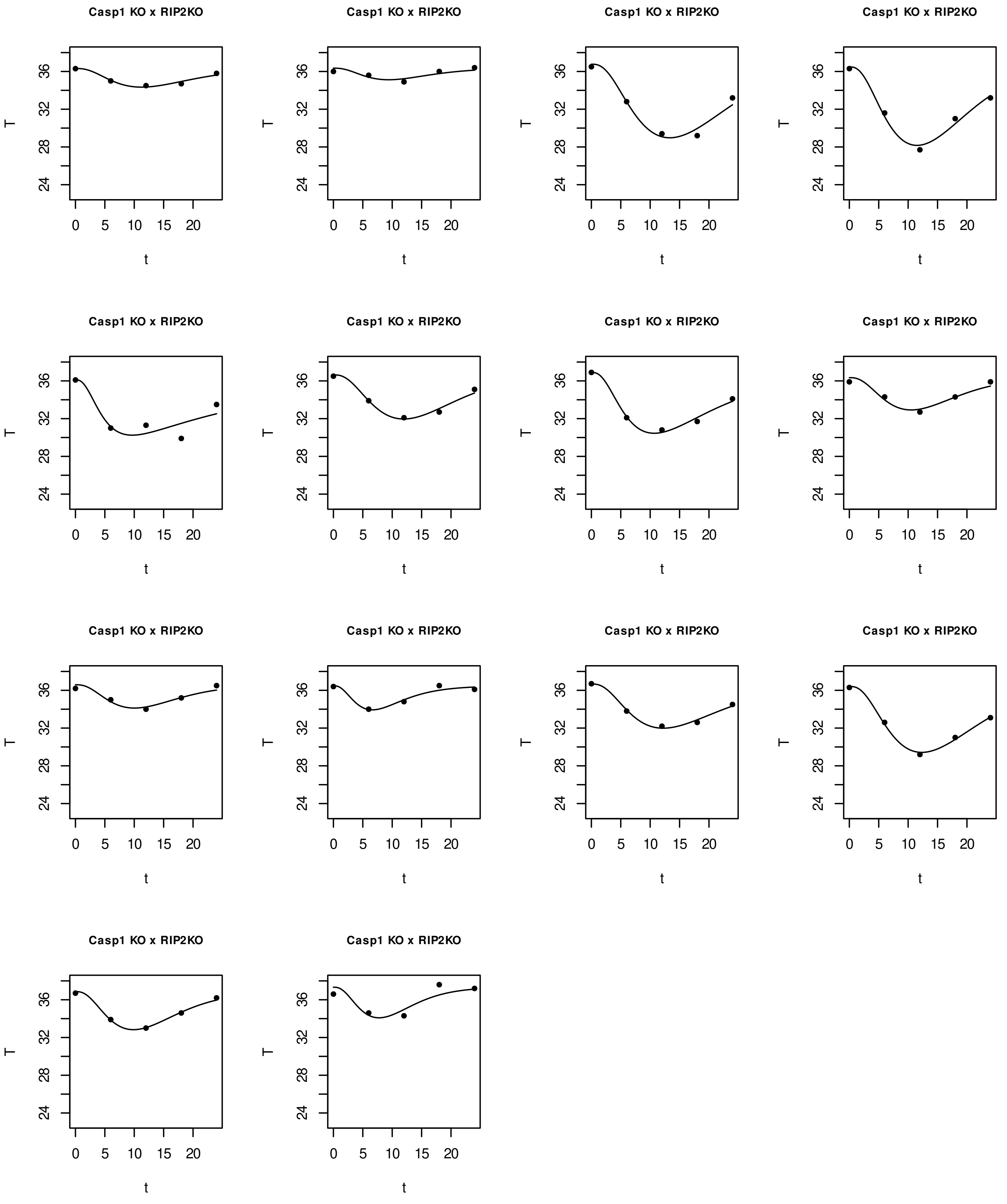
Casp1 KO x RIP2KO
T
t
24
28
32
36
0
5
10
15
20

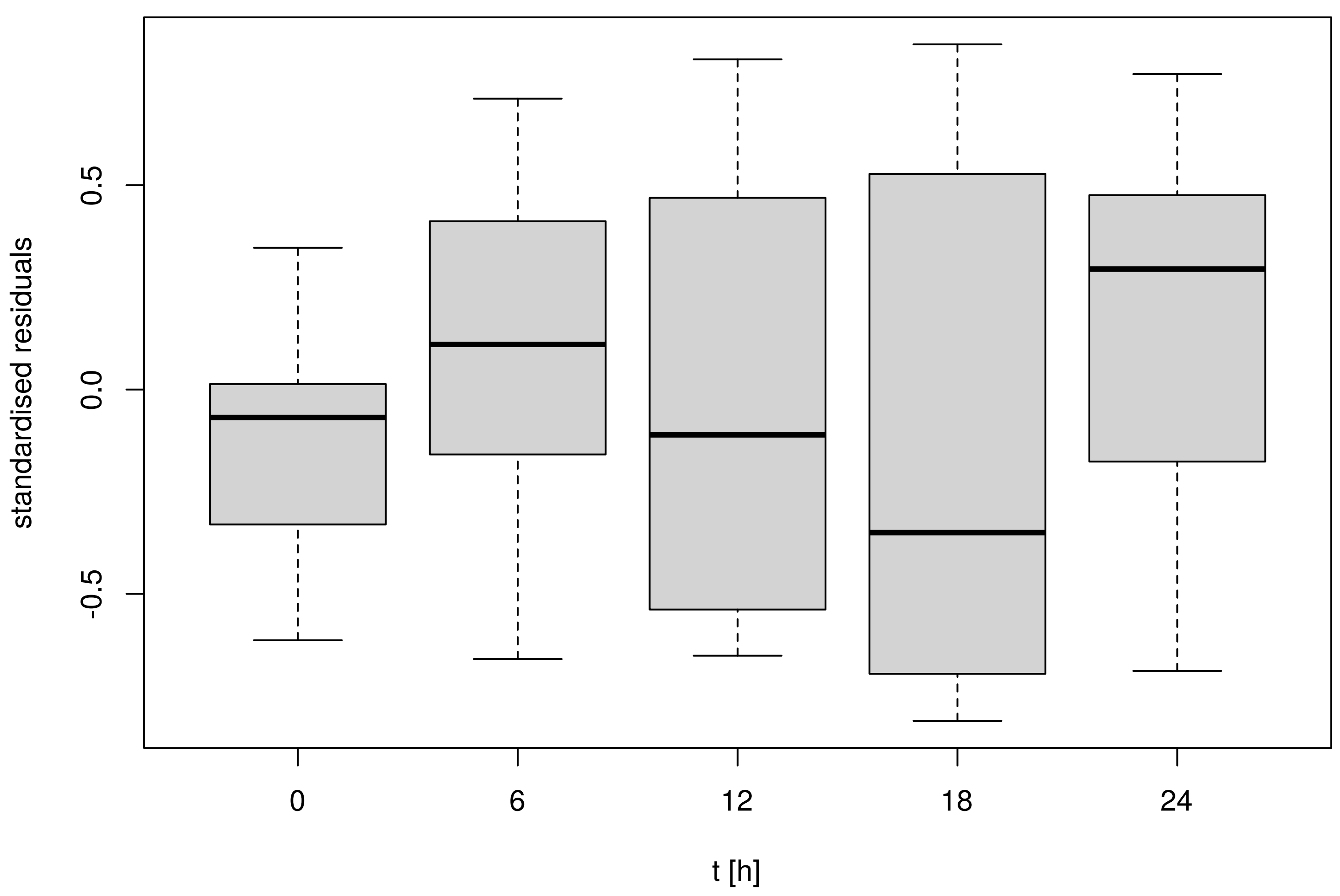
standardised residuals
0.5
0.0
-0.5
0
6
12
18
24
t [h]
